# Twisted atomic magnetic tunnel junctions with multiple nonvolatile states

**DOI:** 10.1038/s41467-026-70239-z

**Published:** 2026-03-12

**Authors:** Yuliang Chen, Kartik Samanta, Alexander J. Healey, Chi Fang, Haojie Zhang, Naafis A. Shahed, David A. Broadway, Arthur Ernst, Evgeny Y. Tsymbal, Stuart S. P. Parkin

**Affiliations:** 1 Max Planck Institute of Microstructure Physics, Halle, Germany; 2 Department of Physics and Astronomy & Nebraska Center for Materials and Nanoscience, University of Nebraska–Lincoln, Lincoln, NE USA; 3 Department of Physics and Materials Science & Engineering, Jaypee Institute of Information Technology, Noida, India; 4 Department of Physics, School of Science, RMIT University, Melbourne, VIC Australia; 5 Institute of Theoretical Physics, Johannes Kepler University, Linz, Austria; 6 Donostia International Physics Center, Donostia-San Sebastian, Spain

**Keywords:** Spintronics, Magnetic devices

## Abstract

Magnetic tunnel junctions (MTJs), a key spintronic device, have shown rapid development recently using two-dimensional (2D) magnets. In particular, MTJs formed from twisted 2D antiferromagnets (AFMs) push nonvolatile magnetic information storage to the atomic limit. Here we demonstrate 2D twisted MTJs with multiple distinct nonvolatile states. Asymmetric MTJ structures formed by twisting a single ferromagnetic CrSBr monolayer and a single antiferromagnetic CrSBr bilayer exhibit two distinct states with up to 700% tunneling magnetoresistance in zero magnetic field at 2 K. By adding a second CrSBr monolayer to form a second twisted interface, four nonvolatile states can be accessed in zero magnetic field. More importantly, any one state among the four states can be switched to any other using magnetic fields. We further demonstrate all-antiferromagnetic MTJs with three twisted antiferromagnetic CrSBr bilayers that exhibit multiple nonvolatile states. Our work shows that it is possible to store multiple-state magnetic information in a single device by integrating several twisted interfaces in the atomic limit.

## Abstract (web summary)

Layered van der Waals magnets like CrSBr, when sandwiched between electrodes form a natural magnetic tunnel junction, albeit lacking the characteristic nonmultiple volatile states at zero field of a magnetic memory. Here, Chen et al overcome this limitation to establish four nonvolatile states at zero field by constructing magnetic tunnel junctions out of twisted monolayers and bilayers of CrSBr with two twisted interfaces.

## Introduction

**Fig. 1:**
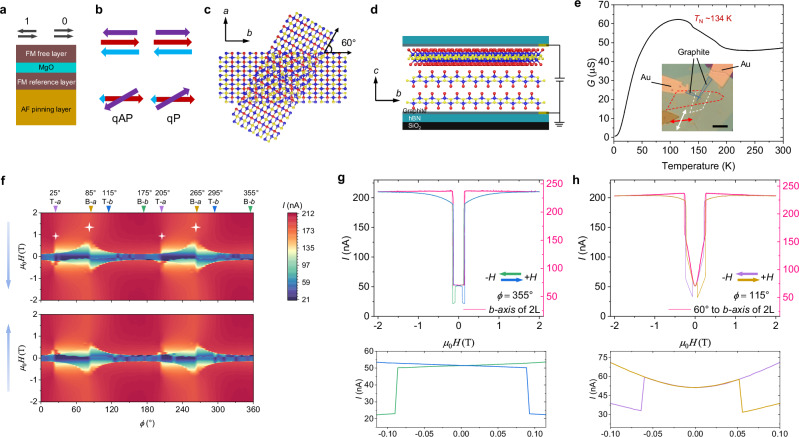
Schematic of a twisted CrSBr bilayer/monolayer MTJ and in-plane magneto-transport results. **a** Structure of conventional MTJs. **b** Two spin configurations with distinct magnetoresistances based on layer-dependent magnetism of a twisted CrSBr 2L/1L structure. qAP: quasi-antiparallel, qP: quasi-parallel. **c** Top view of twisted CrSBr 2L/1L. Blue, yellow, and red balls correspond to Cr, S, and Br, respectively. The *a*, *b* crystal axes of the bottom bilayer are shown. **d** Schematic of a twisted CrSBr 2L/1L MTJ. The stack of **c** (side view here) is sandwiched between thin graphite contacts. The *b*, *c* crystal axes of the bottom bilayer are indicated. **e** Conductance *versus* temperature at ZF. The kink at ~134 K indicates the Néel temperature. Inset, an optical image of the device. The red and white dashed curves outline the bottom bilayer and top monolayer CrSBr flakes, respectively, and the *a*-axis of each flake is indicated by the arrows with the corresponding colors. The blue curves outline the graphite contacts. Scale bar, 5 μm. **f** Field orientation dependence of the tunneling current for field oriented within the *ab* plane. ±2 T field sweep range and 20 mV DC bias are used. Two blue arrows indicate the sweeping direction of the field, backward sweeping for the top panel and forward sweeping for the bottom panel. The inverted triangles with angles mark the position of the crystal axes. T-*a*: the *a*-axis of the top monolayer flake, B-*b*: the *b*-axis of the bottom bilayer flake, etc. The stars mark the humps related to the uniaxial magnetic anisotropy of the CrSBr monolayer and bilayer. **g** Extracted tunneling current *versus* field with field oriented along the *b*-axis of the bottom bilayer flake from (**f**) together with the corresponding results (pink curve) measured on a single CrSBr bilayer MTJ (Supplementary Fig. 1b). The arrows denote the sweeping directions of the field. The lower inset shows a close-up near ZF. **h** Analagous to (**g**), but orienting the field along the *b*-axis of the top monolayer flake (equivalent to 60° to the *b*-axis of the bilayer).

MTJ based reading devices have been used in magnetic disk drives for many years. Due to their excellent scalability, low power consumption, and high endurance, MTJ nonvolatile memory elements are also now used in magnetic random-access memory (MRAM)^1,2^ foundry chips. Furthermore, MTJs have much potential for other applications, such as reading elements in Racetrack Memory^3,4^ and for neuromorphic computing devices^5–8^. The classic MTJ structure (Fig. 1a) comprises a free ferromagnetic (FM) metallic layer and a reference FM metallic layer separated by an ultrathin nonmagnetic oxide insulator, typically MgO^9–13^. In addition, an exchange bias field supplied by an additional antiferromagnetic (AF) layer may be used to pin the magnetization of the reference FM layer (Fig. 1a)^14–16^. Thus, two resistive states (1 or 0) of the MTJ are accessed by flipping the magnetization of the free layer, that is, between an antiparallel configuration (⇆) and a parallel configuration (⇉) of the free and reference FM layers. These two configurations can exhibit very different tunnel resistance states characterized by a large tunneling magnetoresistance (TMR).

Recently explored two-dimensional (2D) magnetic materials^17–19^ are attractive for novel MRAM applications down to the atomic (monolayer, 1L) limit. A 2D bilayer (2L) formed from two monolayers, such as CrI_3_, CrCl_3_, CrPS_4_, and CrSBr^17,20–24^, each with intralayer FM coupling and interlayer AF coupling, exhibits an antiparallel spin configuration in zero field (ZF) and a parallel spin configuration in a large enough field to overcome the interlayer AF coupling. Two nonmagnetic conducting electrodes that sandwich such a magnetic bilayer, that is semiconducting or insulating, thereby form an atomic MTJ (Supplementary Fig. 1a), in which the magnetic bilayer serves as a spin-dependent tunneling barrier distinct from the traditional MTJ structure (Fig. 1a). Giant TMR effects have been demonstrated in 2D MTJs using an external field to switch the spin configuration between antiparallel and parallel alignments^25–36^. Such a bilayer can be taken as the elementary barrier in a multiple-layered architecture, in which the spin configuration at each interface contributes to the final TMR. For example, a trilayer contains two interfaces each with antiparallel aligned moments (top-left diagram in Fig. 1b), resulting in a large tunneling resistance, in contrast to a lower tunneling resistance for the parallel  spin alignment of the top interface (top-right diagram in Fig. 1b). Nonetheless, only the configuration with all antiparallel spin alignments is energetically favorable at ZF on account of the strong interlayer AF coupling in natural 2D AFMs. This, thus, gives rise to volatility at ZF in all 2D MTJs built from natural 2D AFMs^25–35^.

Very recently, 2D MTJs have been shown to exhibit two distinct nonvolatile magnetoresistive states at ZF and multistep magnetization switching at finite fields by twisting two CrSBr bilayers (twisted 2L/2L)^37^ or two CrSBr monolayers (twisted 1L/1L)^37,38^. In such 2D twisted MTJs, the inherent interlayer AF coupling is largely suppressed at the twisted interface, and the significant in-plane magnetic anisotropy of the CrSBr monolayer innately produces a pinning effect. Here, we show that the two nonvolatile states derived from one twisted interface in the twisted CrSBr MTJs can be further extended to multiple nonvolatile states by adding additional twisted interfaces.

As distinct from the previous symmetric twisted 2L/2L and 1L/1L structures^37^, in this report, we first present a twisted atomic MTJ with an asymmetric structure, i.e., by twisting a CrSBr monolayer on top of a natural (untwisted) CrSBr bilayer (twisted 2L/1L, bottom panel of Fig. 1b). In this twisted 2L/1L MTJ, besides the pinning strength derived from the uniaxial in-plane magnetic anisotropy of CrSBr as in the symmetric structures, the interlayer AF exchange interaction in the untwisted CrSBr bilayer provides an additional strong pinning strength, both of which together make the magnetic moments in the untwisted bilayer firmly pinned, thereby resulting in even stronger bistable states in ZF compared with the symmetric structures. Then, we integrate an additional CrSBr monolayer underneath the twisted 2L/1L structure to form a twisted monolayer/bilayer/monolayer (1L/2L/1L) structure. This structure has two twisted interfaces so that four nonvolatile states are created in ZF. Remarkably, due to the substantially increased pinning strength, the four nonvolatile states can be arbitrarily accessed by operating the spin configurations at the two twisted interfaces. Moreover, we further create two twisted interfaces by twisting three AF CrSBr bilayers to develop an all-AF MTJ^37^ with multiple nonvolatile states. Our work sheds light on developing exotic MRAM by taking advantage of not only the atomic layer dimensions of 2D magnets but also multiple storage states in a single device^39,40^.

## Results

### Concept of nonvolatile twisted MTJ

CrSBr is an A-type AF van der Waals (vdW) *n*-type semiconductor with a paramagnetic (PM)-to-AF transition at ~130 K (Néel temperature, *T*_N_) and a substantial intralayer FM coupling evolving at a characteristic higher temperature (*T*_C_, ~150 K)^22,23,41,42^. CrSBr crystallizes in the orthorhombic space group *Pmmn* with crystal axes *a* ≠ *b* ≠ *c* and exhibits a strong magnetocrystalline anisotropy, such, within the *ab* plane, the easy axis lies along the *b*-axis and the hard axis is aligned along the *a-*axis. The uniaxial in-plane magnetic anisotropy and intralayer FM coupling are robust down to the monolayer limit, and the interlayer AF coupling is robust down to the bilayer limit^42–44^. The semiconducting properties and unique magnetic characteristics make CrSBr an ideal platform for exploring 2D twisted MTJs^37,38,45–47^.

The top panel of Fig. 1b shows a 3-layered stack that can be viewed as an assembly of a natural CrSBr bilayer (blue and red arrows) and a top CrSBr monolayer (purple arrow), in which two interfaces are formed. By setting the top monolayer at an angle relative to the bottom bilayer, a top twisted interface can be formed with a twist angle, *θ*_twist_ (bottom panel of Fig. 1b). At ZF the spin configuration is always antiparallel in the lower untwisted interface because of the strong interlayer AF exchange interaction from the linear superexchange chain of Cr-Br-Br-Cr across the vdW gap in the natural CrSBr system^37^. By contrast, the spin configuration at the upper twisted interface has bistable states at ZF, quasi-antiparallel (qAP) and quasi-parallel (qP), due to the negligible magnetic coupling across the twisted interface since a twist with a large *θ*_twist_ considerably tilts the Cr-Br-Br-Cr superexchange chain^37^. Meanwhile, a twist with a large *θ*_twist_ can suppress the formation of moiré superlattices observed in twisted magnets with small *θ*_twist_ (usually less than 10°)^48–52^. The prefix “quasi” denotes that the parallel and antiparallel configurations are not aligned perfectly but rather are misaligned via an acute angle (*θ*_twist_ for qP) and an obtuse angle (π − *θ*_twist_ for qAP). The final tunneling conductance (*G*) of this twisted 3-layered stack is proportional to the product of electron transmissivity (*T*_i_) at each interface ($G\propto {T}_{1}{T}_{2}$) due to a spin-filtering mechanism^37,53^, whose magnitude is related to the relative angle (*θ*_i_) of the spins at the *i*_th_ interface, ${T}_{i}={T}_{{{{\rm{P}}}}}{\cos }^{2}\frac{{\theta }_{i}}{2}+{T}_{{{{\rm{AP}}}}}{\sin }^{2}\frac{{\theta }_{i}}{2}$, where *T*_AP_ and *T*_P_ are the electron transmissivity for perfectly antiparallel and parallel spin alignments, respectively. Accordingly, the two spin configurations in Fig. 1b are expected to have different *G* at ZF, i.e., ZF nonvolatility (ZF NV), even if the spin configuration is always antiparallel in the bottom natural bilayer.

### Twisted CrSBr 2L/1L MTJ

Figure 1c shows a schematic (top view) of a twisted CrSBr 3-layered stack with *θ*_twist_ = 60°, which (side view in Fig. 1d) is further sandwiched by two thin graphite electrodes that are crossed to form a vertical tunneling junction (Fig. 1d). The sandwich structure is further encapsulated by two hBN flakes to protect against degradation in the ambient atmosphere. The inset in Fig. 1e shows an optical image of a fabricated device. The bottom and top flakes are identified by the red and white dashed curves, respectively, and the *a*-axis of each flake is indicated by the arrows. The temperature-dependent *G* at ZF of the twisted 2L/1L MTJ is plotted in Fig. 1e, which displays an overall semiconducting behavior with *G* decreasing with decreasing temperature. At *T* ~ 134 K, a kink appears, manifesting the PM-to-AF transition of the bottom natural CrSBr bilayer in the twisted 3-layered stack. *G* shows a local maximum adjacent to this phase transition temperature due to reduced scattering caused by spin fluctuations as CrSBr becomes magnetically ordered^43^.

When an external magnetic field is applied, the spins in the twisted 3-layered stack will be reoriented, which can be investigated by measuring the tunneling current between the two graphite electrodes. Figure 1f shows the tunneling current as the direction of the field is rotated within the *ab* plane for forward and backward sweeping of the field between ±2 T at 2 K. The inverted triangles at the top of Fig. 1f indicate the positions of the *a* and *b* axes of the top and bottom flakes. These data can be compared with those for a single natural CrSBr bilayer device (Supplementary Fig. 1b). In this way, we find that the characteristics of the natural CrSBr bilayer are well preserved in the twisted 3-layered stack with negligible influence from the top CrSBr monolayer. For example, in Supplementary Fig. 1b, it is observed that the saturation field manifests a hump that gradually decreases when the direction of the external field is rotated from the *a*-axis to the *b*-axis because of the uniaxial magnetic anisotropy, which also appears in Fig. 1f (large stars) but superposed on a tiny hump (small stars). The tiny hump precisely appears at the position of the *a*-axis of the top CrSBr monolayer, which suggests that the uniaxial magnetic anisotropy in the CrSBr monolayer remains robust in the twisted structure without any noticeable influence from the bottom bilayer. We also have measured a single CrSBr monolayer device to confirm the robust uniaxial magnetic anisotropy in the monolayer limit (Supplementary Fig. 2). The above results support the magnetic decoupling at the twisted interface instead of possible interactions modifying the original magnetic anisotropy. Both the CrSBr monolayer and CrSBr bilayer in the twisted 3-layered stack each conserve their uniaxial magnetic anisotropy regardless of the twisted alignment, thereby resulting in a two-fold rotational symmetry in Fig. 1f. Note that all the field-direction-dependent transport measurements in this study adopt a local coordinate (*Φ*) of the measurement system (see Methods), and all magneto-transport experiments were performed at 2 K, except as otherwise noted.

To further inspect the magnetization process in the twisted 2L/1L MTJ upon sweeping the external field, results taken along the *b*-axis of the bottom bilayer (at *Φ* = 355°) are shown together with the corresponding results measured on the single natural bilayer device (pink curve extracted from Supplementary Fig. 1b) in Fig. 1g. For the backward field sweeping case (green curve), when the field decreases to a critical value of ~0.15 T, a steep drop suddenly appears in the tunneling current curve because of the parallel to antiparallel switching of the spin configuration in the bottom CrSBr bilayer since this critical field (*H*_c_) is in good accordance with that of the spin-flip in the single natural bilayer device (pink curve). On further increasing the field along the negative direction to ~−0.16 T, the inverse case of antiparallel to parallel switching also takes place, manifesting a sudden jump in the tunneling current. In contrast to a plateau between 0.15 to −0.16 T in the case of the single natural bilayer device, an extra steep drop emerges at ~−0.086 T (lower inset of Fig. 1g), which is ascribed to the spin-flip transition in the top CrSBr monolayer. Due to hysteresis, a symmetric drop emerges at ~0.086 T for the forward sweeping field curve (blue curve). In Fig. 1h, we plot the case of *Φ* = 115° along the *b*-axis of the top monolayer, in which it is found the spin-flip happens at lower fields of ~±0.053 T in the top monolayer due to the uniaxial magnetic anisotropy (lower inset of Fig. 1h). For the same reason but reversed, the spin-flip in the bottom bilayer happens at larger fields of ~±0.24 T, which also matches the corresponding *H*_c_ measured on the single bilayer device (pink curve in Fig. 1h).

**Fig. 2:**
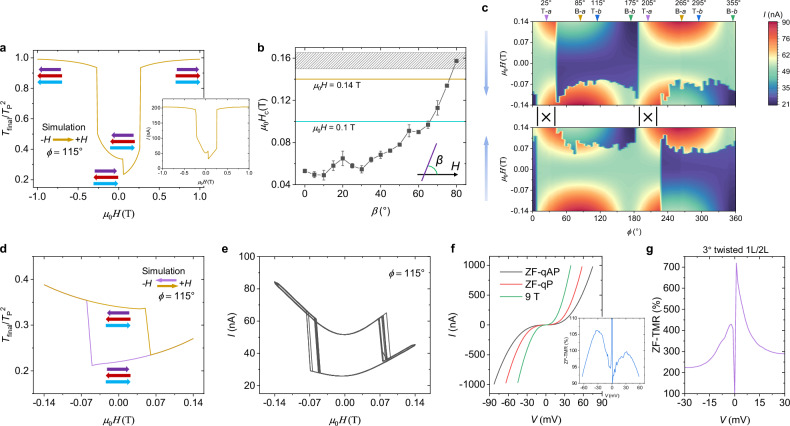
Bistable states at ZF in a twisted CrSBr bilayer/monolayer MTJ. **a** Simulated conductance, $G\propto {T}_{{final}}$, when sweeping field from −1 T to 1 T along the *b*-axis of the top monolayer flake. *T*_P_ is the electron transmissivity for perfectly parallel spin alignment. Inset of the main panel shows the magnetization process, see Supplementary Movie 2 and the description in Supplementary Information. Right inset shows the experimental result extracted from Fig. 1h. **b** Black dots are the extracted critical field (*H*_c_) from Fig. 1f, at which spin-flip happens in the top CrSBr monolayer. *β* is the angular deviation to the *b*-axis (purple line) of the top monolayer, see inset. The filled pattern shows the minimal *H*_c_ of the spin-flip in the natural CrSBr bilayer extracted from Fig. 1f and Supplementary Fig. 1b when sweeping field along its *b*-axis. The gold and cyan lines denote two representative field sweep amplitudes in our experiments. The error bar and width of the filled pattern denote the range of the extracted *H*_c_ owing to hysteresis and thermal fluctuations. **c** Field orientation dependence of the tunneling current for field oriented within the *ab* plane. ±0.14 T field sweep range and 20 mV DC bias are used. At the positions marked by “|x|”, no bistable states appear at ZF. **d** In analogy to (**a**), but dual sweeping field between ± 0.14 T. **e**, Experimental results of tunneling current *versus* sweep field between ±0.14 T for 10 successive loops along the *b*-axis of the top monolayer flake. **f** *I*-*V* curves at ZF and 9 T. Inset, calculated ZF-TMR ratio as a function of bias based on the ZF *I*-*V* curves of ZF-qP *versus* ZF-qAP. **g** ZF-TMR ratio of a 3° twisted device.

Moreover, our micromagnetic simulations nicely reproduce the magnetization processes reflected by the tunneling current measurements of Fig. 1g, h (see Supplementary Movies 1 and 2 and descriptions in Supplementary Information). By further substituting the relative angles between the spins at each of the two interfaces obtained from the simulations into the aforementioned equation, ${T}_{i}={T}_{{{{\rm{P}}}}}{\cos }^{2}\frac{{\theta }_{i}}{2}+{T}_{{{{\rm{A}}}}{{{\rm{P}}}}}{\sin }^{2}\frac{{\theta }_{i}}{2}$, we can qualitatively estimate the tunneling conductance as $G\propto {T}_{{final}}=\,{T}_{1}{T}_{2}$ in Fig. 2a and Supplementary Fig. 4b, d, well reproducing the results of Fig. 1g, h. Note that we do not consider any coupling at the twisted interface, that is, the monolayer and bilayer are isolated in the micromagnetic simulations (see Methods).

### Robust nonvolatility in twisted 2L/1L MTJ

Interestingly, for several *Φ*, we observe two tunneling current states at ZF (i.e., ZF NV) consistent with the schematic in Fig. 1b, but this NV does not appear stably (Supplementary Fig. 3). Furthermore, for most *Φ* in Fig. 1f, only one tunneling current is observed at ZF. The reason is that such major loops^54^ of ±2 T sweep field range flip not only the magnetization of the top monolayer between → and ← but also the magnetization of the bottom bilayer between ⇄ and ⇆ instead of a pinned configuration as indicated by Fig. 1b and see Supplementary Movies 1 and 2 and descriptions in Supplementary Information. If a spin configuration is fixed in the bottom CrSBr bilayer at ZF, the experimentally observed ZF NV can be reproduced by the micromagnetic simulations (Supplementary Figs. 3 and 4).

The above results strongly suggest that we need to reduce the amplitude of the field sweep (i.e., minor loop)^54^ to better pin the spin configuration in the bilayer. From Fig. 1f and Supplementary Fig. 1b, we find that the smallest *H*_c_ to flip the spins in the natural CrSBr bilayer is ~0.15 T when the field is along the *b*-axis (the filled pattern in Fig. 2b). We further extract the *H*_c_ *versus* angle relative to the *b*-axis (*β*, inset of Fig. 2b) that flip the spin in the CrSBr monolayer from Fig. 1f. It is found that *H*_c_ increases with *β* (Fig. 2b) resulting from the uniaxial in-plane magnetic anisotropy that persists even to the monolayer limit.

Figure 2b shows that for fields less than 0.15 T the spin configuration in the bottom CrSBr bilayer can be well pinned at ZF but that the spin orientations in the top CrSBr monolayer can be switched, which is first confirmed by the simulations in Fig. 2d and Supplementary Fig. 5. Then, more convincingly, the field-direction-dependent transport measurements in Fig. 2c display ZF NV over extensive angular *Φ* ranges except for the angular ranges marked by “|x|” since the ±0.14 T sweep field range we used is not enough to flip the spin in the CrSBr monolayer near its *a*-axis (see Fig. 2b). The observed ZF NV is very stable, as checked by 10 hysteresis loops at different *Φ* (Fig. 2e and Supplementary Fig. 6). Figure 2b also reveals that the range of *Φ* where the ZF NV appears will shrink when the amplitude of the sweeping field is reduced, which is validated by the experiments in Supplementary Fig. 7. In the previous discussion, we argued that the flipped spin configurations in the bottom bilayer result in volatility at ZF using the major loops, which is further confirmed by the mirrored tunneling current curves^55^ in Supplementary Fig. 6l-n. Such minor loop results are obtained after a large field stimulation flips the spin configuration to its time-reversal copy in the bottom bilayer. The mirrored results are also reproduced by simulations (Supplementary Fig. 5).

Next, we investigate the performance of the twisted CrSBr 2L/1L MTJ. Figure 2f is the *I*-*V* feature of the binary states at ZF, labeled “ZF-qAP” and “ZF-qP” in line with Fig. 1b for the low-conductance state and high-conductance state, respectively. The *I*-*V* curve at 9 T is also presented for comparison, whose tunneling current is the largest because the spins in the twisted 3-layered stack are all perfectly parallel at 9 T. The inset of Fig. 2f shows the ZF-TMR ratio between “ZF-qAP” and “ZF-qP”, which reaches up to 100%. A more than 130% ZF-TMR ratio is realized in another 45° twisted 2L/1L MTJ (Supplementary Fig. 9). In a 3° twisted 1L/2L MTJ, the ZF-TMR is even higher, up to 700% (Fig. 2g and Supplementary Fig. 10). Note that the 3° twisted 1L/2L MTJ has the top bilayer and bottom monolayer. The experimental TMR agrees with the conclusion in ref. ^37^, that is, a smaller *θ*_twist_ favors a larger TMR. Moreover, analogous behaviors are also reproduced in the 3° twisted 1L/2L MTJ (Supplementary Fig. 10), which strongly indicates that the formed moiré superlattice with a small *θ*_twist_ (Supplementary Fig. 10a) does not produce a strong interlayer coupling but instead decoupling at the twisted interface in the CrSBr system, as also validated by our density functional theory (DFT) calculations (see Supplementary Table 1 and Methods). More remarkably, the high-temperature measurements demonstrate that the ZF NV is robust close to *T*_N_ but a single state at ZF once the temperature is above *T*_N_ (Supplementary Figs. 8 and 11). Thus, nonvolatile 2D MTJs with an asymmetric structure are successfully achieved.

The ZF NV in the twisted CrSBr 2L/1L MTJs is observed over almost all *Φ* except for the angular ranges marked by “|x|” (Fig. 2c, Supplementary Figs. 7, 9b and 10d). In comparison, the ZF NV only appears in narrow angular *Φ* ranges in the symmetric structures of twisted CrSBr 2L/2L MTJs and twisted CrSBr 1L/1L MTJs, see ref. ^37^. This is because, in addition to the in-plane uniaxial magnetic anisotropy which is the only source for pinning in the symmetric twisted structures^37^, the twisted 2L/1L structure has an additional pinning strength derived from the interlayer AF exchange interactions in the bottom untwisted bilayer relative to the top monolayer.

### Four states in twisted 1L/2L/1L MTJ

**Fig. 3:**
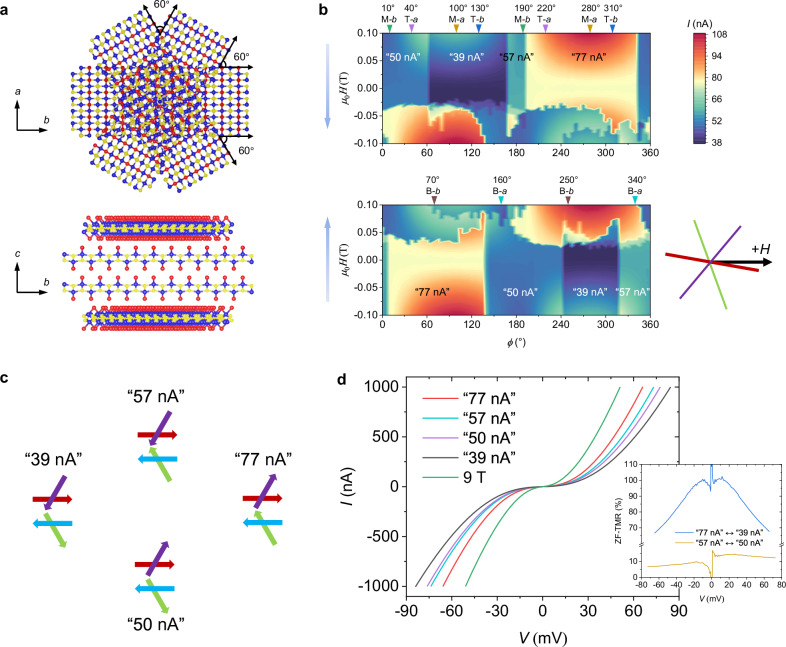
Four states at ZF in a twisted CrSBr monolayer/bilayer/monolayer MTJ. **a** Schematic of twisted CrSBr monolayer/bilayer/monolayer. Top view in the upper panel and side view in the lower panel. *a*, *b*, *c* crystal axes of the mid bilayer are indicated. **b** Field orientation dependence of the tunneling current for field oriented within the *ab* plane. ±0.1 T field sweep range and 20 mV DC bias are used. Two blue arrows indicate the sweeping direction of the field, backward sweeping for the top panel and forward sweeping for the bottom panel. Inverted triangles with angles mark the position of the crystal axes. T-*a*: the *a*-axis of the top monolayer flake, M-*b*: the *b*-axis of the mid bilayer flake, B-*a*: the *a*-axis of the bottom monolayer flake and so on. We label the four nonvolatile states with each ZF current. Inset shows the external field (*H*) direction relative to the easy axes of the three CrSBr flakes when *Φ* = 0°. Purple, red and green lines correspond to the easy axes of the top, mid and bottom flakes, respectively. **c** Four spin configurations correspond to the different tunneling currents at ZF. Purple arrow for the spin in the top monolayer, red and blue arrows for the spins in the mid bilayer, and green arrow for the spin in the bottom monolayer. **d** Four *I*-*V* curves at ZF and one at 9 T. Inset, calculated ZF-TMR ratios as a function of bias based on the ZF *I*-*V* curves of “77 nA” *versus* “39 nA” and “57 nA” *versus* “50 nA”.

As shown in Fig. 1c, d, we misaligned the top CrSBr monolayer to form one twisted interface in the twisted 2L/1L MTJs. Next, we further extend this concept by misaligning an additional CrSBr monolayer under the bilayer to form a second twisted interface. In this twisted 4-layered stack (Fig. 3a), 60° twist angles are used, and note the relative misaligned angle is also 60° between the top and the bottom monolayers (top view, upper panel of Fig. 3a). To better exhibit the spin configurations at the three interfaces of the twisted 4-layered stack, we redraw a diagram based on Fig. 1b but shift the arrows only for clarity, as shown in Fig. 3c, in which the spin in the bottom monolayer is denoted by a green arrow. Now, the natural CrSBr bilayer is the mid-flake with an antiparallel spin configuration (the red and blue arrows in Fig. 3c). There are four different spin configurations in the twisted 4-layered stack because of either the qP or the qAP spin arrangement at each of the two twisted interfaces.

The twisted 2L/1L in Fig. 1d is replaced with the 4-layered stack in Fig. 3a to form a twisted 1L/2L/1L MTJ. Supplementary Fig. 12a shows the field-direction-dependent tunneling currents with the field swept between ±2 T, which have similar characteristics as Fig. 1f except for an additional hump appearing at the right side of the large hump due to the bottom CrSBr monolayer. Again, we do not observe any stable ZF NV if the applied magnetic fields are too large. Figure 3b shows the field-direction-dependent tunneling currents by sweeping the field between ±0.1 T. As distinct from the bistable states in Fig. 2c and Supplementary Figs. 9b and 10d, four states appear at ZF in Fig. 3b. We label the four ZF states with each tunneling current value at ZF (the measurement error is <1 nA). The four ZF states correspond to the four spin configurations in Fig. 3c. If the spins are in qAP (qP) alignment at both twisted interfaces, the tunneling current is the lowest (largest). For the other two cases with qAP alignment at one twisted interface and qP alignment at the other twisted interface, ideally they should display an identical moderate tunneling current, while the asymmetries introduced via device fabrication, such as nonidentical twist angles and strains, cause different tunneling currents in a practical situation (Supplementary Fig. 19). Note that the corresponding relationship between ZF states and spin configurations in Fig. 3c is strictly linked, which will be discussed later. The *I*-*V* curves of the four ZF states and the 9 T state are plotted in Fig. 3d. An up to 100% ZF-TMR ratio is obtained in the interchange between the “77 nA”-state and the “39 nA”-state. The interchange between the “57 nA”-state and the “50 nA”-state gives the lowest ZF-TMR ratio but it is still more than 10%, and the additional ZF-TMR ratios for the other cases are given in Supplementary Fig. 12c.

### Manipulation among the four states

**Fig. 4:**
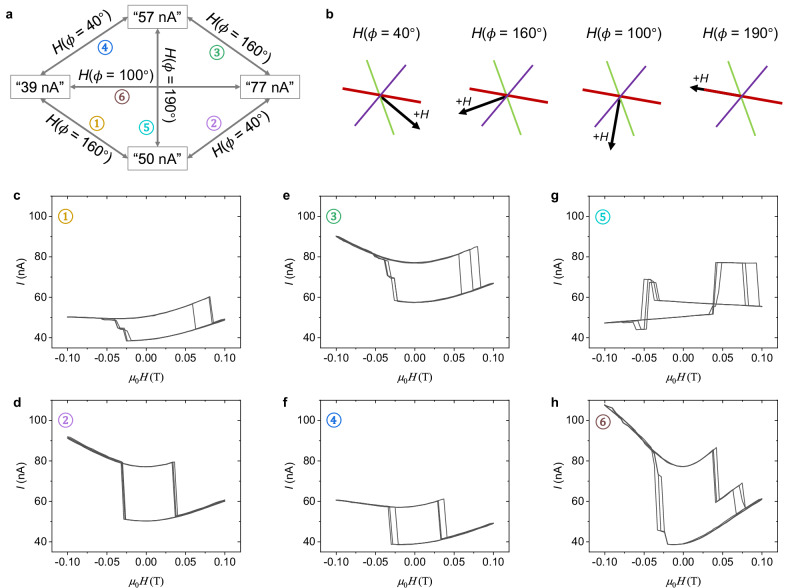
Manipulating the four nonvolatile states in the twisted CrSBr monolayer/bilayer/monolayer MTJ. **a** Diagram of interchange relationships among the four states connected by *H*(*Φ*). *H*(*Φ*) means the external field is oriented at *Φ*. There are six interchanges, which are numbered. **b** Analagous to the inset of Fig. 3b, but with different *Φ*. **c**–**h** Experimental demonstrations of the interchange relationships in (**a**). Three successive loops are used for each interchange. ±0.1 T field sweep range and 20 mV DC bias are used.

The inset in Fig. 3b shows the external field direction relative to the easy axes of the three CrSBr flakes in the twisted 4-layered stack when *Φ* = 0°, and Fig. 3b shows that rotating the orientation of the field sweep determines which two of the four ZF states in the twisted 1L/2L/1L MTJ can be interchanged. For example, the “77 nA” -states can be turned to the “50 nA”-state and the “39 nA”-state by forward sweeping the field and returning to ZF at 10° <*Φ* < 60° and 60° <*Φ* < 135°, respectively. We now further investigate the interchange principle. First, we extract four representative operations (see Fig. 4b; *H*(*Φ*) means the external field is oriented at *Φ*) that are closely related to the crystal axes of the three CrSBr flakes in the twisted 1L/2L/1L MTJ by recapping the results of Figs. 3b, 2c and Supplementary Figs. 9b and 10d. The spin configuration in the mid CrSBr bilayer is constantly pinned owing to the ±0.1 T field sweep range. Hence, to switch the four ZF states, one only needs to manipulate the spins in the top and bottom monolayers. See Fig. 4b, *H*(*Φ* = 40°) is oriented along the *a*-axis of the top monolayer, whose spin is pinned owing to the strong uniaxial magnetic anisotropy (see Fig. 2b). However, *H*(*Φ* = 40°) corresponds to a 30° deviation to the *b*-axis of the bottom monolayer (see Fig. 4b), so a ±0.1 T field is enough to flip the spin in the bottom monolayer as indicated by Fig. 2b. It is found in Fig. 3c that solely manipulating the spin in the bottom monolayer can realize two interchanges, that are, “57 nA”-state ↔ “39 nA”-state and “50 nA”-state ↔ “77 nA”-state, which is established by the experimental results (Fig. 4d, f). Based on the same principle, *H*(*Φ* = 160°) solely manipulates the spin in the top monolayer to realize the interchanges of “57 nA”-state ↔ “77 nA”-state and “50 nA”-state ↔ “39 nA”-state (Fig. 4c, e). Figure 4a summarizes the interchange relationships among the four ZF states, which suggests that we can set the device to any of the four states and even indirectly achieve the interchanges of the “50 nA”-state ↔ “57 nA”-state and  the “39 nA”-state ↔ “77 nA”-state by combining the *H*(*Φ* = 160°) and *H*(*Φ* = 40°) operations to switch the spins in the top/bottom monolayers subsequently.

In addition, direct interchanges of “50 nA”-state ↔ “57 nA”-state and “39 nA”-state ↔ “77 nA”-state can be established if an operation can simultaneously switch both the spins in the top/bottom monolayers. Figure 3c shows that the relative angle of the spins in the top/bottom monolayers is always 120° but in opposite orientations for the “50 nA”-state and the “57 nA”-state, i.e., the spins in the top/bottom monolayers of the “50 nA”-state (“57 nA”-state) point to the right (left) of the *b*-axis of the mid CrSBr bilayer. We further note that the *b*-axis of the mid bilayer is the angular bisector of this 120° relative angle, see *H*(*Φ* = 190°) in Fig. 4b, which corresponds to a 60° deviation from both the *b*-axes of the top/bottom monolayers. Accordingly, per Fig. 2b, a ±0.1 T field is sufficient to simultaneously operate the two spins in the top/bottom monolayers using *H*(*Φ* = 190°) to enable a direct interchange of “50 nA”-state ↔ “57 nA”-state. Based on the same analysis, *H*(*Φ* = 100°) enables a direct interchange of “39 nA”-state ↔ “77 nA”-state. Both direct interchanges are experimentally verified (Fig. 4g, h). These interchanges are also shown in Fig. 4a.

## Discussion

The four operations in Fig. 4a have distinct properties. The operations of *H*(*Φ* = 100°) and *H*(*Φ* = 190°) are independent of the initial state. For example, if the initial state is set at “57 nA” which is not relevant to the operation of *H*(*Φ* = 100°) as seen in Fig. 4a, *H*(*Φ* = 100°) can still make the interchange back to “39 nA”-state ↔ “77 nA”-state (Supplementary Fig. 13). Accordingly, *H*(*Φ* = 100°) and *H*(*Φ* = 190°) can be exploited as an initialization operation. By contrast, the initial state determines which states will be accessed by the operations of *H*(*Φ* = 40°) and *H*(*Φ* = 160°). For example, *H*(*Φ* = 40°) switches the state to “39 nA” instead of the others if preparing the initial state as “57 nA” (Fig. 4a). This is one reason that we can precisely assign the tunneling currents to the spin configurations, as shown in Fig. 3c. Another reason is that if we apply a large field stimulation to flip the spin configuration in the mid CrSBr bilayer (⇄, see Fig. 3c) to its time-reversal copy as ⇆, the tunneling current curves are mirrored (Supplementary Fig. 14).

We reproduce four ZF states and interchange relationships in another 60° twisted 1L/2L/1L MTJ (Supplementary Fig. 18) and another twisted 1L/2L/1L MTJ with a 30° twisted top interface and a 60° twisted bottom interface (Supplementary Fig. 19). More remarkably, the high-temperature measurements demonstrate that four ZF states and interchange relationships are retained at 120 K close to *T*_N_ (Supplementary Figs. 15−17), but a single state at 140 K (Supplementary Fig. 15e). To further ensure the four ZF states arising from the four spin configurations of Fig. 3c rather than magnetic domains, we visualize the domain reversal of a 1L device using a cryogenic widefield nitrogen-vacancy diamond microscope^46^, and confirm a single domain emerges around ZF (Supplementary Fig. 20)^56–58^. Note that the magnetoconductance steps adjacent to *H*_c_ due to the formation of domains^38,46^ would not influence the ZF NV (Supplementary Note 1). The concept of creating multiple nonvolatile states via multiple twisted interfaces is further extended to an all-AF MTJ by twisting three AF CrSBr bilayers^37^, which shows three nonvolatile states possibly due to the near tunneling currents of the two spin configurations with qAP alignment at one twisted interface and qP alignment at the other twisted interface (Supplementary Fig. 21). We also perform DFT calculations for the TMR of the 60° twisted interface using the model proposed in ref. ^37^. From the complex band structure of CrSBr, we extract the lowest decay rates in the two-dimensional Brillouin zone (2DBZ; Supplementary Fig. 22) based on which a 93.5% ZF-TMR is obtained, which is in good agreement with the experimentally measured value of ~100% (inset of Fig. 2f).

Finally, we briefly discuss the application prospects of our 2D twisted MTJs. Although we have only demonstrated accessing the multiple NV states via magnetic field, nevertheless, this is an important first step towards the use of such devices technologically. Indeed, the original studies on conventional MTJs involved accessing their states via magnetic fields and it was only later that current-induced spin torques were realized to access these states. Our results establish the possibility of creating multiple nonvolatile states in the twisted 2D vdW MTJs. It is remarkable that individual layers as thin as one vdW unit form almost perfect tunnel barriers, whereas it took decades for the development of conventional tunnel barriers using thin oxide layers that are inevitably replete with defects that severely reduce the spin polarization of the current. The use of ultrathin vdW layers as tunnel barriers, especially in multiple-ground-state MTJs opens the door to the widespread exploration of vdW layers, some of which will be switchable via electric currents or fields and/or optical fields. See Supplementary Note 2 for some further discussion regarding scaling, operation, and miniaturization.

In conclusion, we have explored two types of 2D twisted MTJs with an asymmetric structure, namely twisted CrSBr 2L/1L MTJ and twisted CrSBr 1L/2L/1L MTJ. In the former, two very stable states are created at ZF because of one twisted interface. In the latter, four nonvolatile states are created at ZF because of two twisted interfaces. We have also explored an all-AF MTJ, twisted CrSBr 2L/2L/2L with two twisted interfaces, in which three nonvolatile states are created at ZF. We anticipate that our strategy can be further extended to more nonvolatile states by creating more twisted interfaces in the future.

## Methods

### Device fabrication

We prepared three electrodes on Si/SiO_2_(285 nm) substrates by standard electron-beam lithography (EBL, Raith Pioneer 2), followed by Ti(2 nm)/Au(30 nm) deposition (scia Coat 200) to form the electrodes. Those electrodes are distributed in a “T”-shape. We fabricated the CrSBr MTJs using the same transfer procedures as in ref. ^37^. For some devices, we found that the transferred graphite electrodes were broken after dissolving the residual poly(bisphenol A carbonate), possibly due to strain produced during the transferring procedures^56^. For those devices, backup electrodes were prepared using another EBL procedure followed by Ti/Au deposition in analogy to the “T”-shape electrodes. To better ensure an MTJ works on a single domain at ZF for a large TMR, it is preferred to fabricate the tunneling area as small as possible. Our tunneling areas are less than 1 μm^2^.

### Electrical transport measurements

All electrical measurements were conducted in a PPMS DynaCool cryostat (Quantum Design, Inc) with a base temperature of 1.8 K and magnetic field up to 9 T. The conductivity-temperature results were measured in a two-terminal configuration using a Keithley 2450 source-meter to source a 1 mV DC bias and measure current, a warmup recipe of 1 K/min being used. All magneto-transport experiments were also performed in a two-terminal configuration using a Keithley 2450 source-meter to source a DC voltage and measure current. The DC results were verified by AC measurements using a 7270 DSP lock-in amplifier showing no substantial difference. Note that we were only concerned about the in-plane magneto-transport. The TMR ratio is quantified by (*G*_h_-*G*_l_)/*G*_l_, where *G*_l_ and *G*_h_ are the low conductance and high conductance, respectively. All field-direction-dependent transport measurements adopted the pre-calibrated local coordinates of the PPMS system and the rotational angle is defined as *Φ*. We adopted a unified method to mount the devices onto the electrical transport puck by taking the “T”-distributed Ti/Au electrodes as the reference object. The “|” electrode and the other two “—” electrodes were aligned along the side edge and bottom edge of the puck by hand, respectively, resulting in the direction of the “|” electrode being almost along the direction of the magnetic field when *Φ* = 0°. This unified custom helped us to examine the twist angles and locate the crystal axes of CrSBr flakes. We estimate the uncertainty in angle is ~±5°.

### Micromagnetic and stray field simulations

All micromagnetic simulations were carried out on a micromagnetic module integrated in COMSOL Multiphysics^59,60^. In our simulations, we considered the Landau-Lifshitz-Gilbert (LLG) equation, $\frac{\partial {{{\boldsymbol{m}}}}}{\partial t}=-\gamma {{{\boldsymbol{m}}}}\times {{{{\boldsymbol{H}}}}}_{{{{\boldsymbol{eff}}}}}+\alpha {{{\boldsymbol{m}}}}\times \frac{\partial {{{\boldsymbol{m}}}}}{\partial t}$, where ***m*** is the macrospin vector, ***H***_**eff**_ is the effective field, *γ* is the gyromagnetic ratio, and *α* is the Gilbert damping coefficient. For ***H***_**eff**_ we only included the sources from magnetic anisotropy energy, Zeeman energy, and for the natural CrSBr bilayer the interlayer exchange energy is additionally included. Note that we did not consider any energy at the twisted interface. We ignored other energy sources, such as demagnetization energy, thermal fluctuations. We used a large *α* = 0.5 to accelerate the relaxation process and a long evolutionary time of 20 ns to ensure the whole system reaches an equilibrium. We qualitatively depicted the tunneling conductance as $G\propto {T}_{{final}}=\,{T}_{1}{T}_{2}$, in which ${T}_{i}={T}_{{{{\rm{P}}}}}{\cos }^{2}\frac{{\theta }_{i}}{2}+{T}_{{{{\rm{AP}}}}}{\sin }^{2}\frac{{\theta }_{i}}{2}$. For simplicity, we assumed that the TMR = 150% between the perfectly antiparallel and parallel spin alignments, which means *T*_AP_ = 0.4*T*_P_.$\,{T}_{{final}}$ was normalized in unit of ${T}_{{{{\rm{P}}}}}^{2}$. Since the demagnetization energy favors magnetic domains, we then added it to our simulations, but we find the results are almost identical to the previous ones, which means the demagnetization energy is not the main reason accounting for the magnetic domains near *H*_c_ (Supplementary Note 1)^56^. The stray field simulations were carried out using the magnetostatic module integrated in COMSOL Multiphysics. In the simulations we assume that every Cr carries a 3*μ*_B_ magnetic moment, where *μ*_B_ is the Bohr magneton, thus corresponding to a remanent flux density of 0.2475 T. A 2D geometry was adopted, that is, the *bc* plane of the CrSBr 1L was simulated, and the sample size was set to be 10 nm and 0.834 nm along the *b*- and *c*- directions, respectively.

### Widefield nitrogen-vacancy images

The cryogenic widefield nitrogen-vacancy microscope was integrated into a closed-cycle cryostat with a base temperature of 4 K (AttoDry1000) with a 1 T superconducting vector magnet (Cryomagnetics). Optical control was performed with a 532 nm continuous wave laser (Laser Quantum Ventus 1 W) coupled to a single-mode fiber. A 60 mm collimation lens at the output of the fiber was used to adjust the beam size at the sample. The laser was controlled by an acousto-optical modulator (AAOpto MQ180-G9-Fio) and is directed into the cryostat with a dichroic beam splitter, where it passes through a low-temperature microscope objective (Attocube LT-APO/VISIR/0.82). The nitrogen-vacancy PL is collected by a path that contains a 4 f lens configuration and is separated by the dichroic mirror and a 731/137 nm band-pass filter, before being focused (300 mm tube lens) onto a water cooled sCMOS camera (Andor Sona). The microwave control was performed with a signal generator (Rohde & Schwarz SMB100A), a switch (Mini- Circuits ZASWA-2-50DR+), and a 50 W amplifier (Mini-Circuits HPA-50W-63), which is directed into the cryostat and through a custom-made printed circuit board containing a straight waveguide with a width of 1 mm. The measurements were controlled and synchronized with a pulse pattern generator (SpinCore PulseBlasterESR-PRO 500 MHz). At each applied field, the magnetic field images were projected onto the *b*-axis of the CrSBr sample with a nitrogen-vacancy orientation along the *b*-axis and a tilt of 55° from the *c*-axis. The applied field is along the nitrogen-vacancy axis. Due to spin-mixing in the excited state of the nitrogen-vacancy, there is a gap in the magnetic field data between 35 and 60 mT. See more experimental details in ref. ^46^.

### DFT calculations

The electronic structure and the optimal interlayer separation are carried out based on DFT using the plane-wave projected augmented wave (PAW) method^61^ as implemented in the Vienna ab initio Simulation Package (VASP)^62,63^. We used the Perdew-Burke-Ernzerhof (PBE) exchange-correlation functional^64^ within the generalized gradient approximation (GGA). The electron-electron correlation effects beyond GGA are taken into account using the Hubbard U correction via the GGA + U method^65^ with *U* value of 4.0 eV and Hund’s coupling energy *J* of 0.8 eV on the Cr-3*d* orbitals. For the self-consistent calculations, a plane-wave basis set with a plane-wave cutoff of 500 eV and a *k*-point mesh of 12 × 8 × 4 and 12 × 8 × 1 is used for the CrSBr bulk and monolayer, respectively. The structural optimization is carried out using VASP while maintaining the symmetry of the heterostructure. The positions of the atoms are relaxed toward equilibrium until the Hellman–Feynman forces become less than 0.01 eV/Å.

Calculations of the complex band structure are performed using the non-equilibrium Green’s function formalism (DFT + NEGF approach)^66,67^, as implemented in QuantumATK, Synopsys QuantumATK^68^. In QuantumATK, the nonrelativistic Fritz-Haber-Institute (FHI) pseudopotentials are employed with a single-zeta-polarized basis, and a cutoff energy is set to 150 Ry. The spin-polarized GGA + U method is used in our calculations with the same *U* and *J* values on the Cr-3*d* orbitals as in VASP. *k*-point mesh of 18×16×10 is used for bulk CrSBr. Periodic boundary conditions are assumed for the transverse direction (*x-y* plane) and open boundary conditions along the transport *z*-direction. Since CrSBr is an *n*-type semiconductor, we applied *E*_F_ = *E*_CBM_ − 0.152 eV to match the experimental TMR. A 60° twist angle was used in the non-collinear spin configuration and the coherent tunneling process^37^. The lattice constants of *a* = 3.540 Å, *b* = 4.755 Å and *c* = 8.394 Å for the bulk CrSBr are assumed in the calculations from a database (mp-22998)^69^. The experimentally measured lattice constants^23^ are also substituted into our calculations showing no substantial difference. For simplicity, we neglect any role of the graphite electrodes (note that the graphite lattice is incommensurate with the CrSBr lattice) and assume that they simply provide electronic states for transport that are uniformly distributed in the 2DBZ of CrSBr. The same method in ref. ^37^ was used to calculate the interlayer exchange interactions with 3° and 5° twists. See Supplementary Fig. 23 and Supplementary Table 1, although small rotations reduce the interlayer interactions, they still exist due to the slight tilt of the superexchange chains of Cr–Br–Br–Cr (Supplementary Fig. 23b)^37^. Then we added a small shift of 0.5 Å along the *b*-axis (it is reasonable that a twist consists of a rotation and a shift), and the results are far below that of the untwisted interface due to the shift breaking the superexchange chains (Supplementary Fig. 23c). Supplementary Table 1 also shows that the interlayer distance *c* has negligible impact on the interlayer exchange interactions.

## Supplementary information

Supplementary Information

Peer Review File

Description of Additional Supplementary Files

Supplementary Movie 1

Supplementary Movie 2

## Data Availability

The data that support the findings of this study are available from the corresponding authors upon request. Source data for the main results are available at Zenodo repository^70^ (10.5281/zenodo.18328335).
