# Supplementary material for: Twisted atomic magnetic tunnel junctions with multiple nonvolatile states
Source: Nat Commun. 2026 Mar 12;17:2439. doi: 10.1038/s41467-026-70239-z (PMC12988127; doi:10.1038/s41467-026-70239-z)
Supplement: Supplementary file 1 — Supplementary Information [file 41467_2026_70239_MOESM1_ESM.pdf]

Note added in proof:

Our manuscript has been previously reviewed at another Nature Research journal since 29. 09. 2024 until transferring to Nature Communications.

Contents

Supplementary Figs. 1-23

Supplementary Table 1

Supplementary Notes 1-2

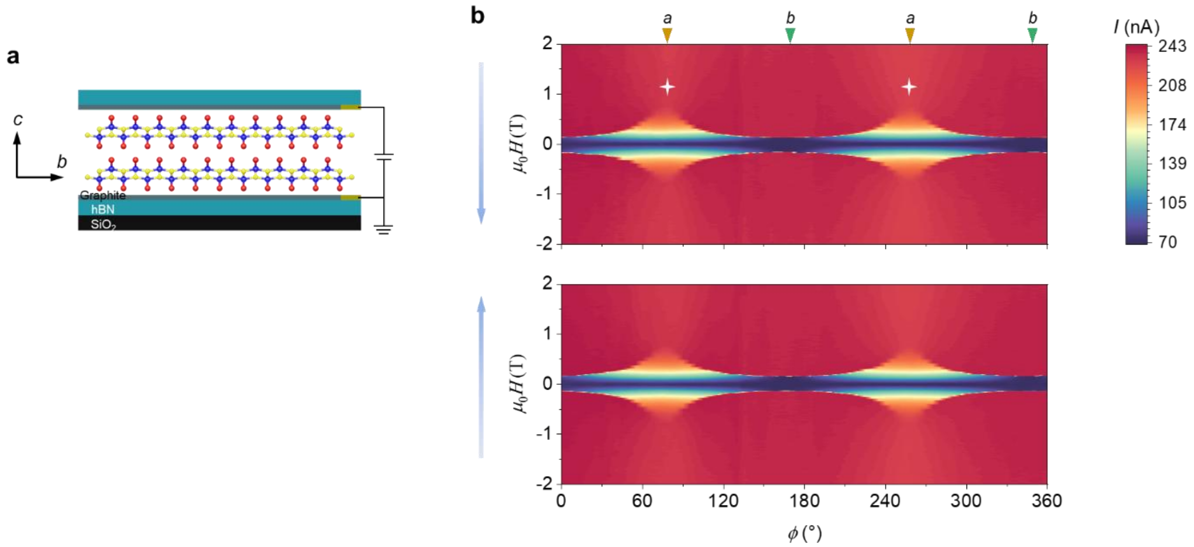

Supplementary Fig. 1 | Schematic of a single natural CrSBr bilayer MTJ and in-plane magneto-transport results. a, Schematic of a single natural CrSBr bilayer MTJ. **b**, Field orientation dependence of the tunneling current for field oriented within the *ab* plane at 2 K. ±2 T field sweep range and 10 mV DC bias are used. Two blue arrows indicate the sweeping direction of the field, backward sweeping for the top panel and forward sweeping for the bottom panel. The inverted triangles mark the position of the crystal axes. The critical field gradually increases with rotating field from *b*-axis to *a*-axis due to the uniaxial magnetic anisotropy of the CrSBr bilayer.

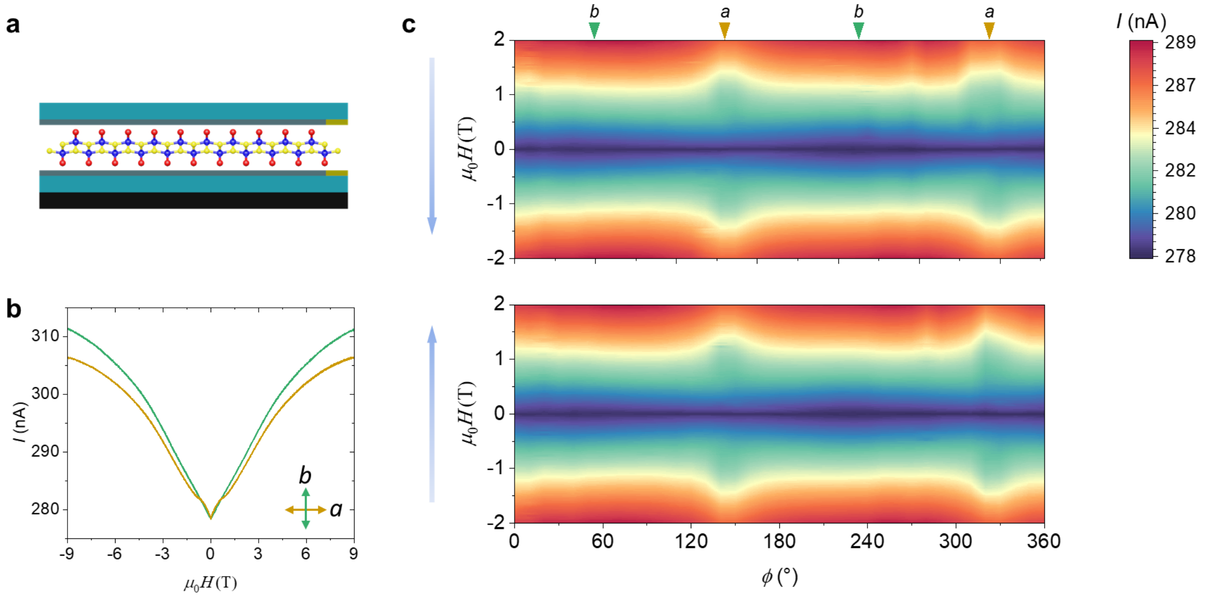

Supplementary Fig. 2 | Schematic of a single CrSBr monolayer device and in-plane magneto-transport results. a, Schematic of a single CrSBr monolayer device. **b**, Tunneling current *versus* field with field oriented along different directions as indicated by the inset. ±9 T field sweep range and 5 mV DC bias are used. **c**, Field orientation dependence of the tunneling current for field oriented within the *ab* plane. ±2 T field sweep range and 5 mV DC bias are used. Two blue arrows indicate the sweeping direction of the field, backward sweeping for the top panel and forward sweeping for the bottom panel. The inverted triangles mark the position of the crystal axes. Despite no tunneling magnetoresistance stemming from the spin-filtering effect as the CrSBr bilayer MTJ in Supplementary Fig. 1, the anisotropic conductance at the strong field regime suggests that the uniaxial magnetic anisotropy is robust to the CrSBr monolayer.

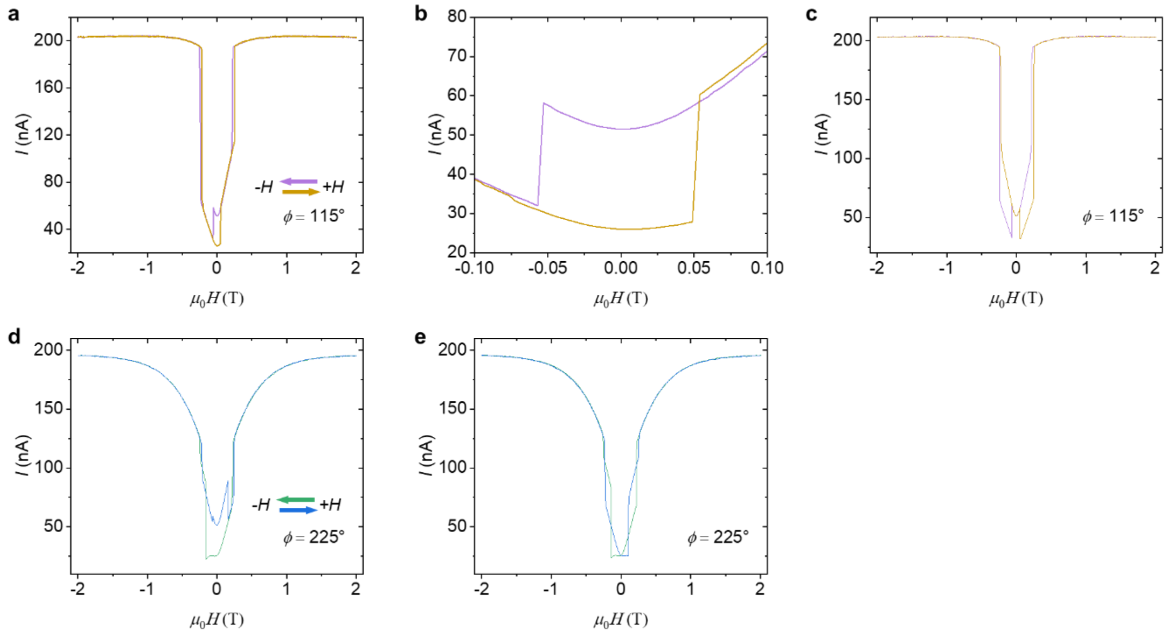

Supplementary Fig. 3 | Unstable ZF NV in the twisted CrSBr 2L/1L MTJ. a-c, Dual sweeping field at Φ = 115°. **b** shows a close-up of **a** near ZF. **d-e**, Dual sweeping field at Φ = 225°. ±2 T field sweep range and 20 mV DC bias are used for **a-e**.

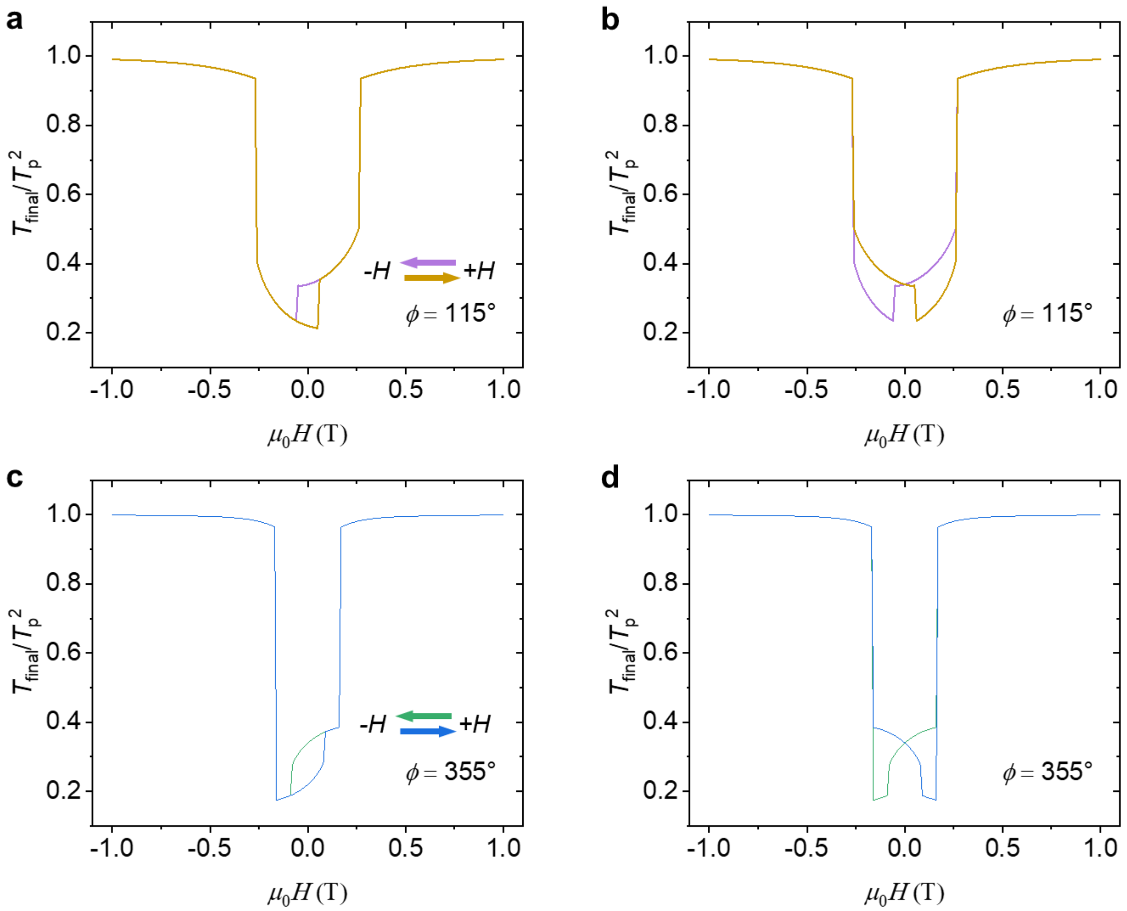

Supplementary Fig. 4 | Simulated conductance, $G \propto T_{final}$, T_P is the electron transmissivity for perfectly parallel spin alignment. a-b, Results of Φ = 115°. **c-d**, Results of Φ = 355°. For **a** and **c**, a fixed spin configuration is preset at ZF in both forward and backward field sweeps. For **b** and **d**, the spin configuration is the time-reversal copy of one another at ZF in forward and backward field sweeps. ±1 T field sweep range is used for **a-d**.

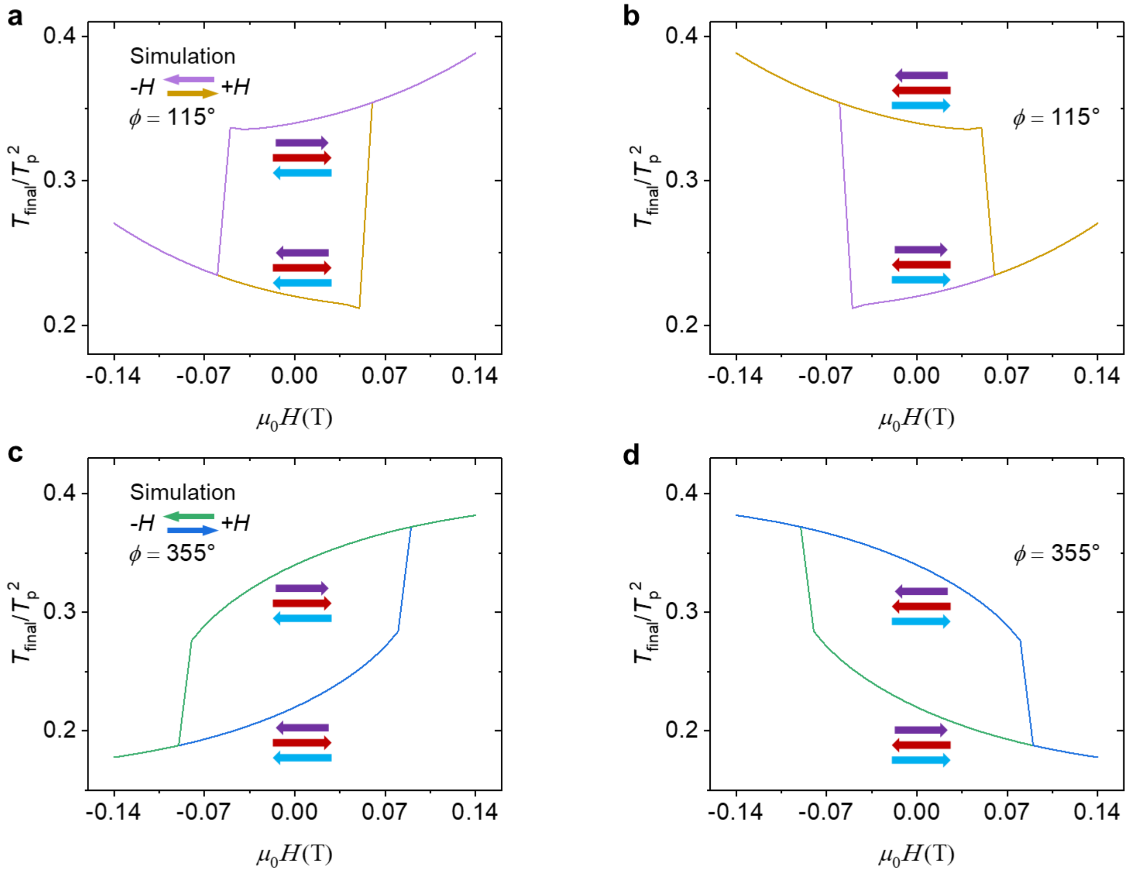

Supplementary Fig. 5 | Simulated conductance, $G \propto T_{final}$, T_P is the electron transmissivity for perfectly parallel spin alignment. a-b, Results of Φ = 115°. **c-d**, Results of Φ = 355°. ±0.14 T field sweep range is used for **a-d**. Insets, spin configurations at ZF. Comparing **a** with **b**, **c** with **d**, the curves are mirrored when the spin configuration becomes its time-reversal copy in the CrSBr bilayer.

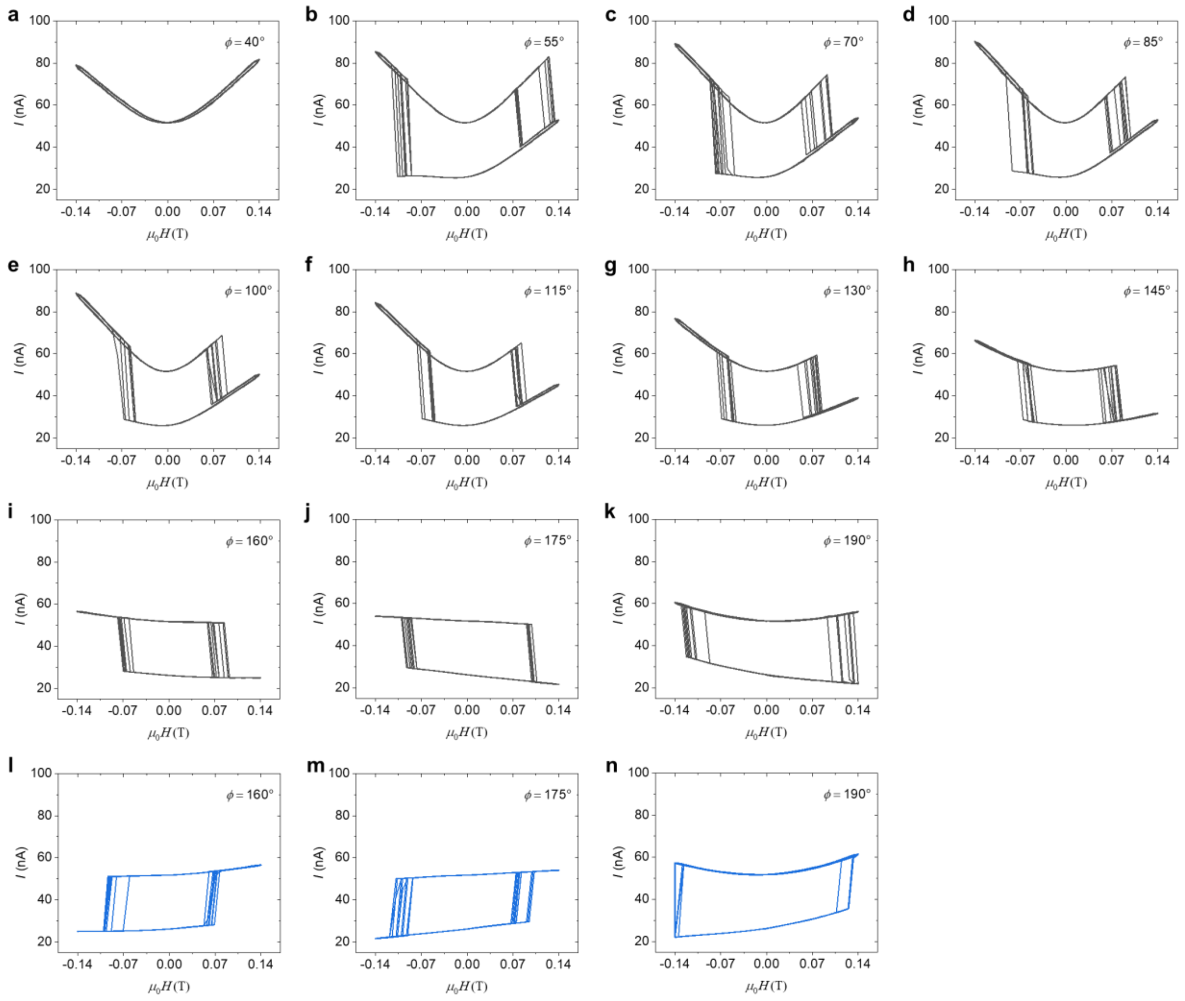

Supplementary Fig. 6 | Stable ZF NV in the twisted CrSBr 2L/1L MTJ. a-n, 10 successive loops of sweeping field between ±0.14 T at each Φ. 20 mV DC bias is used. **l-n** are measured after a strong field stimulation flips the spin configuration to its time-reversal copy in the CrSBr bilayer. Compared **i-k** with **l-n**, the curves are mirrored.

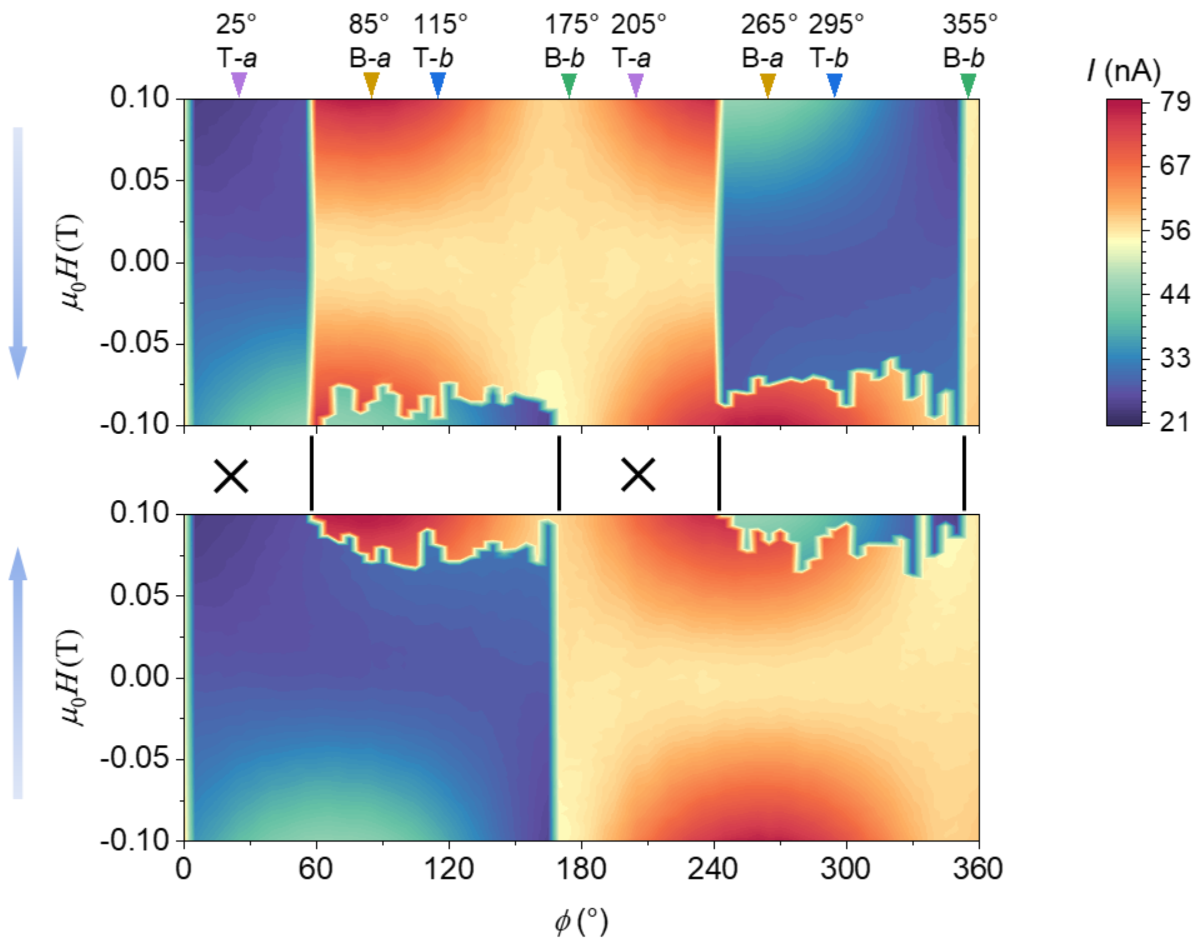

Supplementary Fig. 7 | Additional in-plane magneto-transport results of the twisted CrSBr 2L/1L MTJ. Field orientation dependence of the tunneling current for field oriented within the *ab* plane. ±0.1 T field sweep range and 20 mV DC bias are used. At the marked positions by |×|, no bistable states appear at ZF.

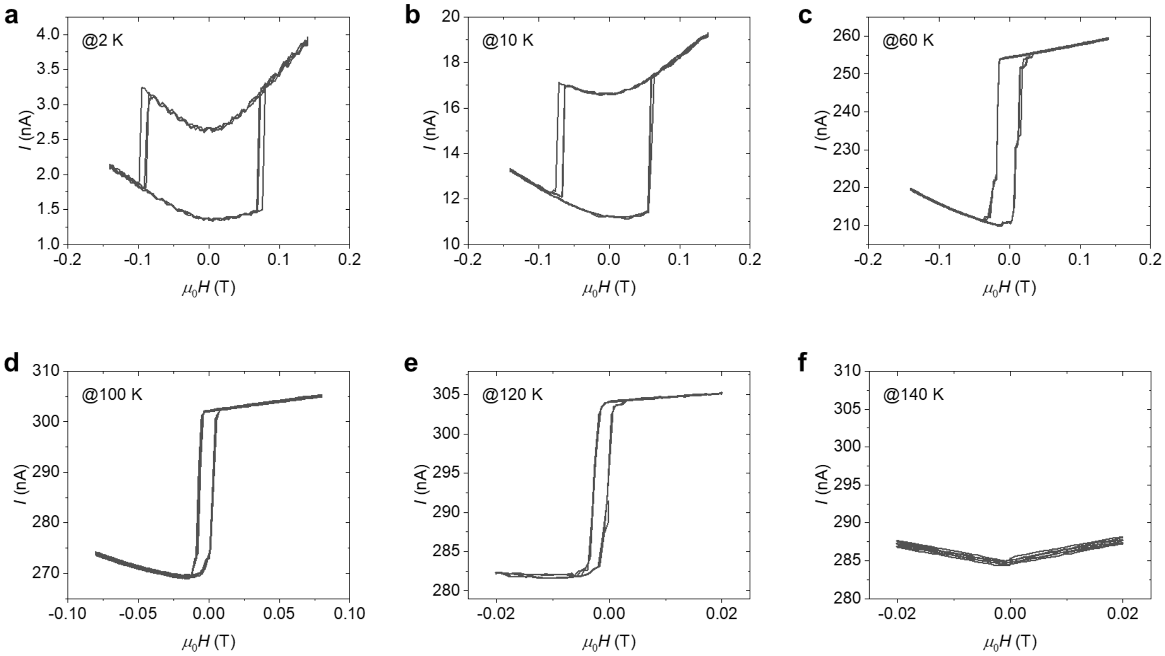

Supplementary Fig. 8 | Temperature dependence of the ZF NV in the twisted CrSBr 2L/1L MTJ. 3 successive loops of sweeping field at each temperature. Φ = 115° and 5 mV DC bias are used.

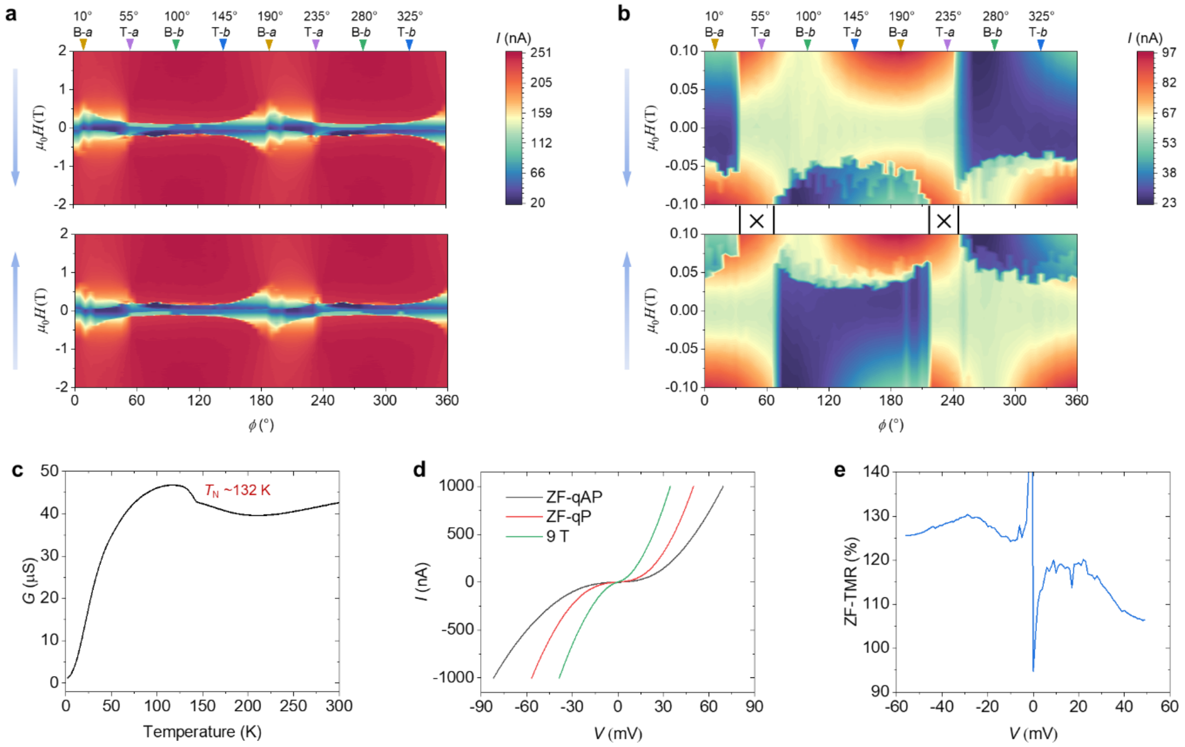

Supplementary Fig. 9 | Experimental results measured on a 45° twisted CrSBr 2L/1L MTJ. a-b, Field orientation dependence of the tunneling current for field oriented within the *ab* plane. ±2 T field sweep range for **a**, ±0.1 T field sweep range for **b**, and 15 mV DC bias for both. **c**, Conductance *versus* temperature at ZF. **d**, *I*-*V* curves at ZF and 9 T. **e**, Calculated ZF-TMR ratio as a function of bias based on the ZF *I*-*V* curves of **d**.

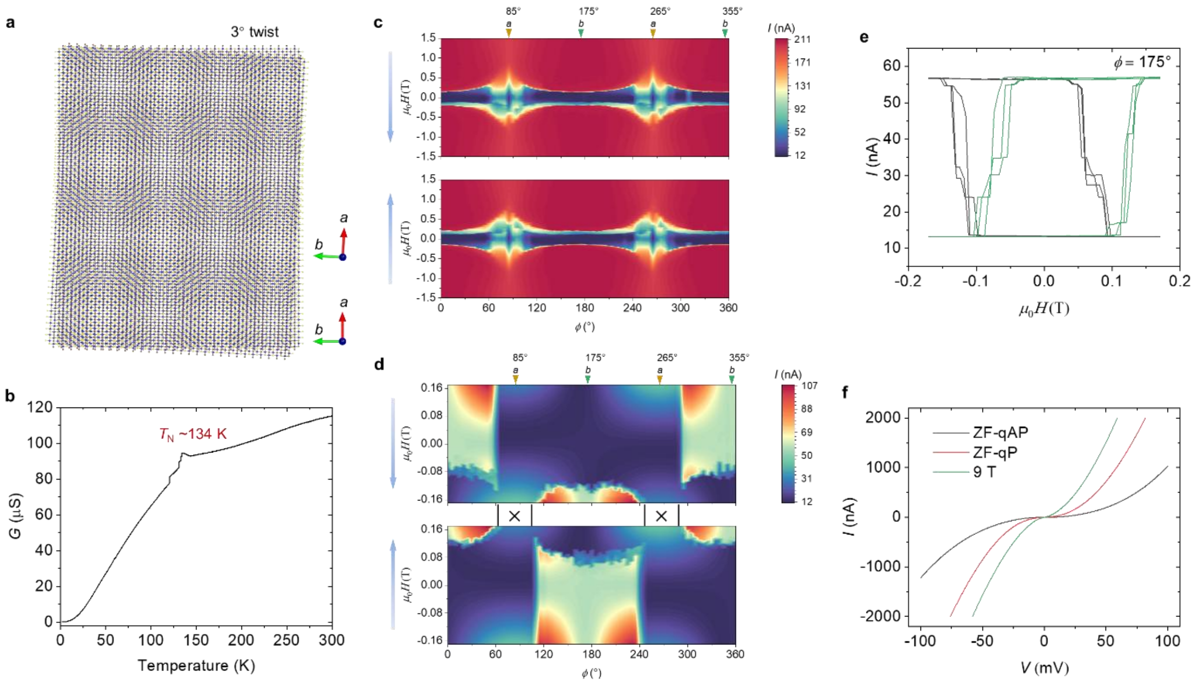

Supplementary Fig. 10 | Experimental results measured on a 3° twisted CrSBr 1L/2L MTJ. a, Top view of 3° twisted CrSBr 1L/2L. **b**, Conductance *versus* temperature at ZF. **c-d**, Field orientation dependence of the tunneling current for field oriented within the *ab* plane. ±1.5 T field sweep range for **c**, ±0.17 T field sweep range for **d**, and 15 mV DC bias for both. **e**, 3 successive loops of sweeping field between ±0.17 T at Φ = 175°. 15 mV DC bias is used. The green and grey curves are mirrored since the magnetic configurations in the 2L are time-reversal of one another. **f**, *I*-*V* curves at ZF and 9 T.

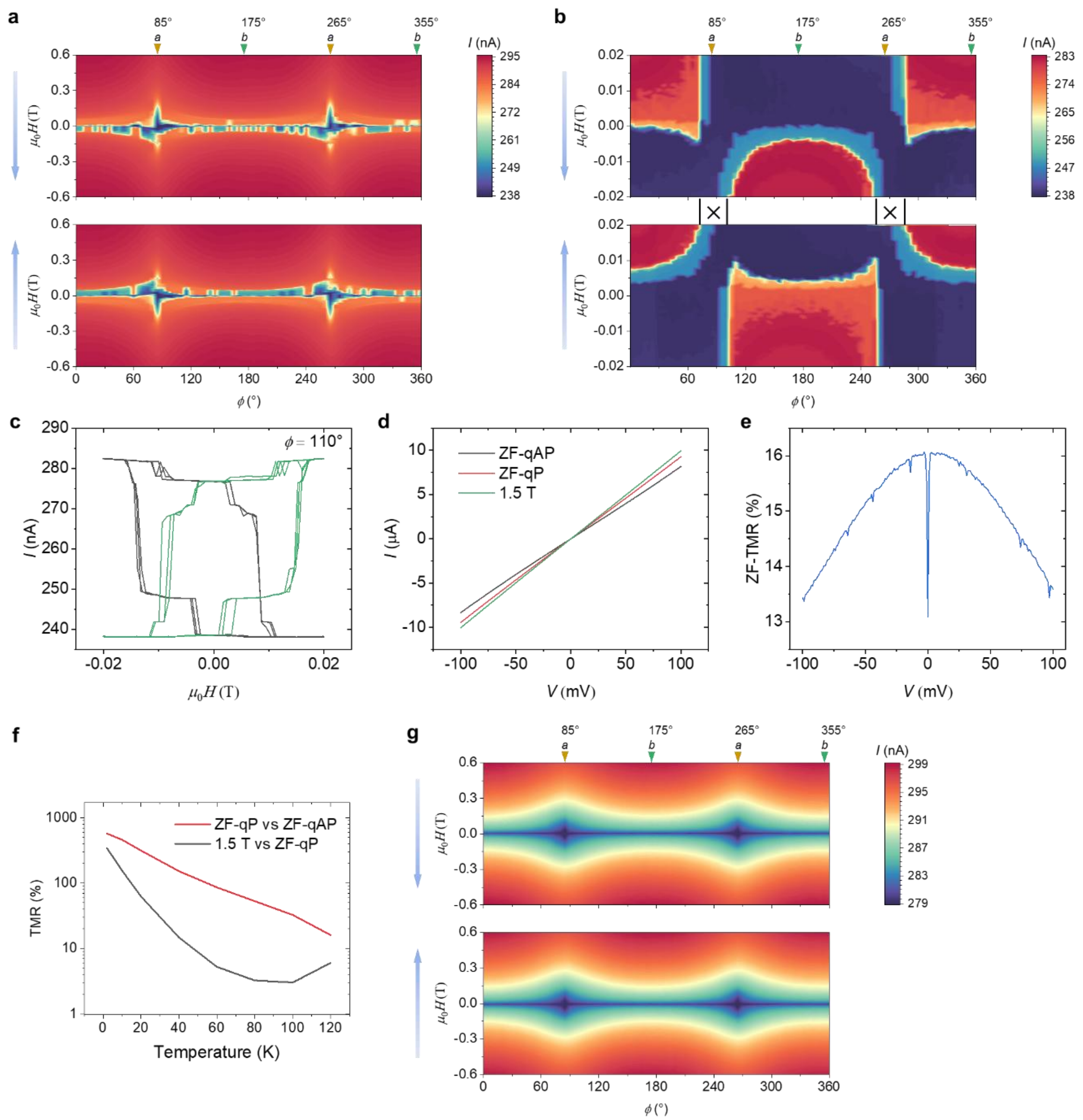

Supplementary Fig. 11 | High-temperature experimental results of the 3° twisted CrSBr 1L/2L MTJ. a-b, Field orientation dependence of the tunneling current for field oriented within the *ab* plane. ±0.6 T field sweep range for **a**, ±0.02 T field sweep range for **b**, and 3 mV DC bias for both. **c**, 3 successive loops of sweeping field between ±0.02 T at Φ = 110°. 3 mV DC bias is used. The green and grey curves are mirrored since the magnetic configurations in the 2L are time-reversal of one another. **d**, *I-V* curves at ZF and 9 T. **e**, Calculated ZF-TMR ratio as a function of bias based on the ZF *I-V* curves of **d**. **a-e** are obtained at 120 K. **f**, TMR ratio as a function of temperature. 3 mV DC bias is used. **g**, Field orientation dependence of the tunneling current for field oriented within the *ab* plane. ±0.6 T field sweep range and 3 mV DC bias are used at 140 K.

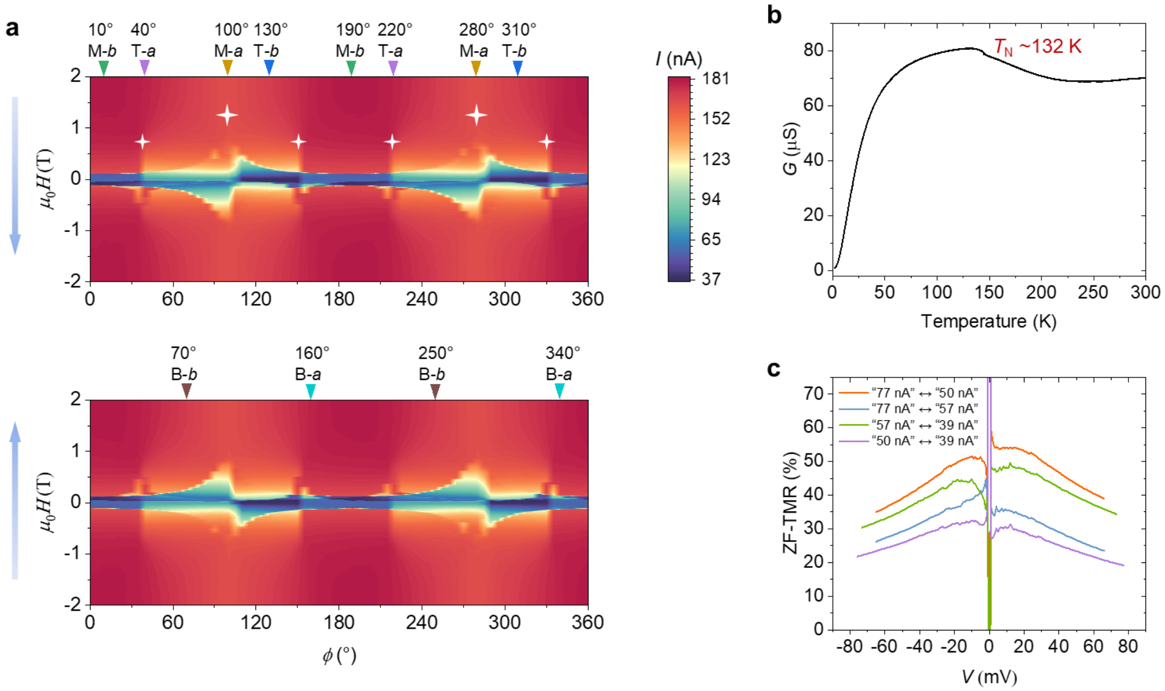

Supplementary Fig. 12 | Additional experimental results of the twisted CrSBr monolayer/bilayer/monolayer MTJ. a, Field orientation dependence of the tunneling current for field oriented within the *ab* plane. ±2 T field sweep range and 20 mV DC bias are used. Two blue arrows indicate the sweeping direction of the field, backward sweeping for the top panel and forward sweeping for the bottom panel. The inverted triangles with angles mark the position of the crystal axes. T-*a*: the *a*-axis of the top monolayer flake, M-*b*: the *b*-axis of the mid bilayer flake, B-*a*: the *a*-axis of the bottom monolayer flake and so on. The stars mark the humps which are related to the uniaxial magnetic anisotropy of the CrSBr monolayers and bilayer. **b**, Conductance *versus* temperature at ZF. **c**, Other calculated ZF-TMR ratios as a function of bias based on the ZF *I*-*V* curves in Fig. 3d of the main text.

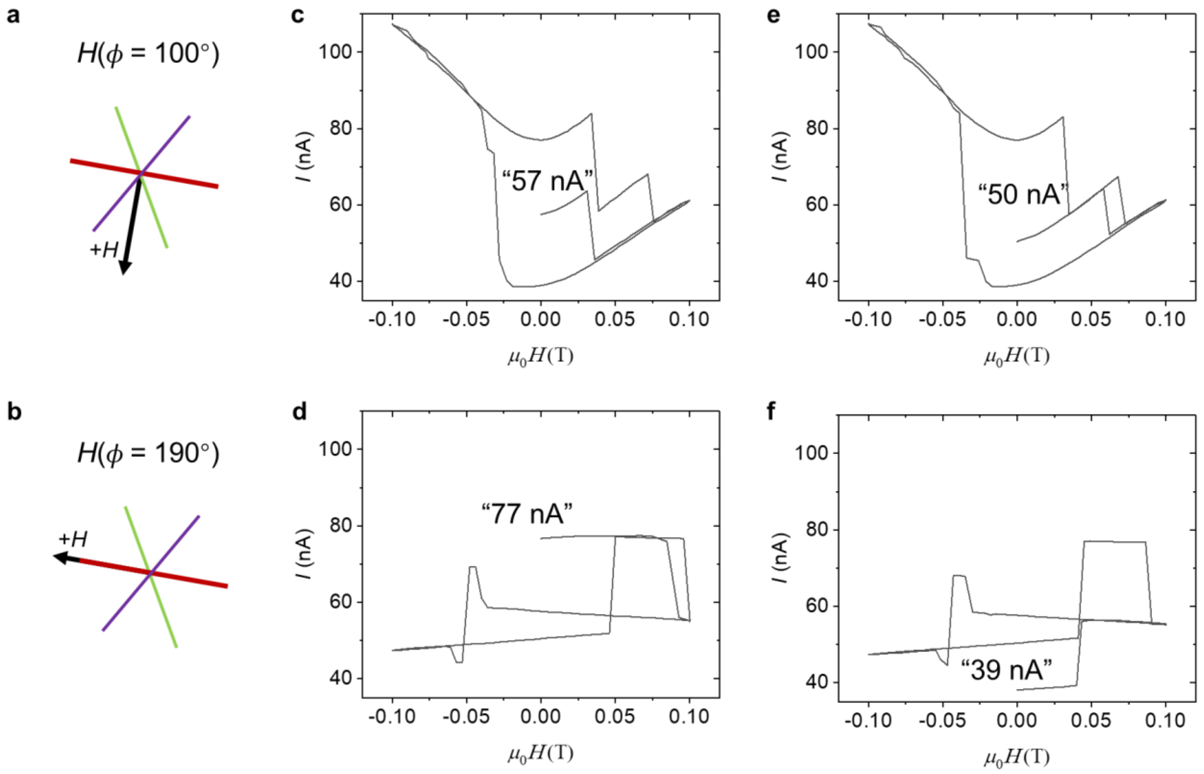

Supplementary Fig. 13 | Demonstrations of initialization by *H*(*Φ* = 100°) and *H*(*Φ* = 190°). a and **b**, Diagram of *H*(*Φ* = 100°) and *H*(*Φ* = 190°), respectively, adapted from Fig. 4b of the main text. **c**-**f**, Experimental demonstrations of initialization by *H*(*Φ* = 100°) and *H*(*Φ* = 190°). *H*(*Φ* = 100°) for **c** and **e**, *H*(*Φ* = 190°) for **d** and **f**. Each initial state is indicated.

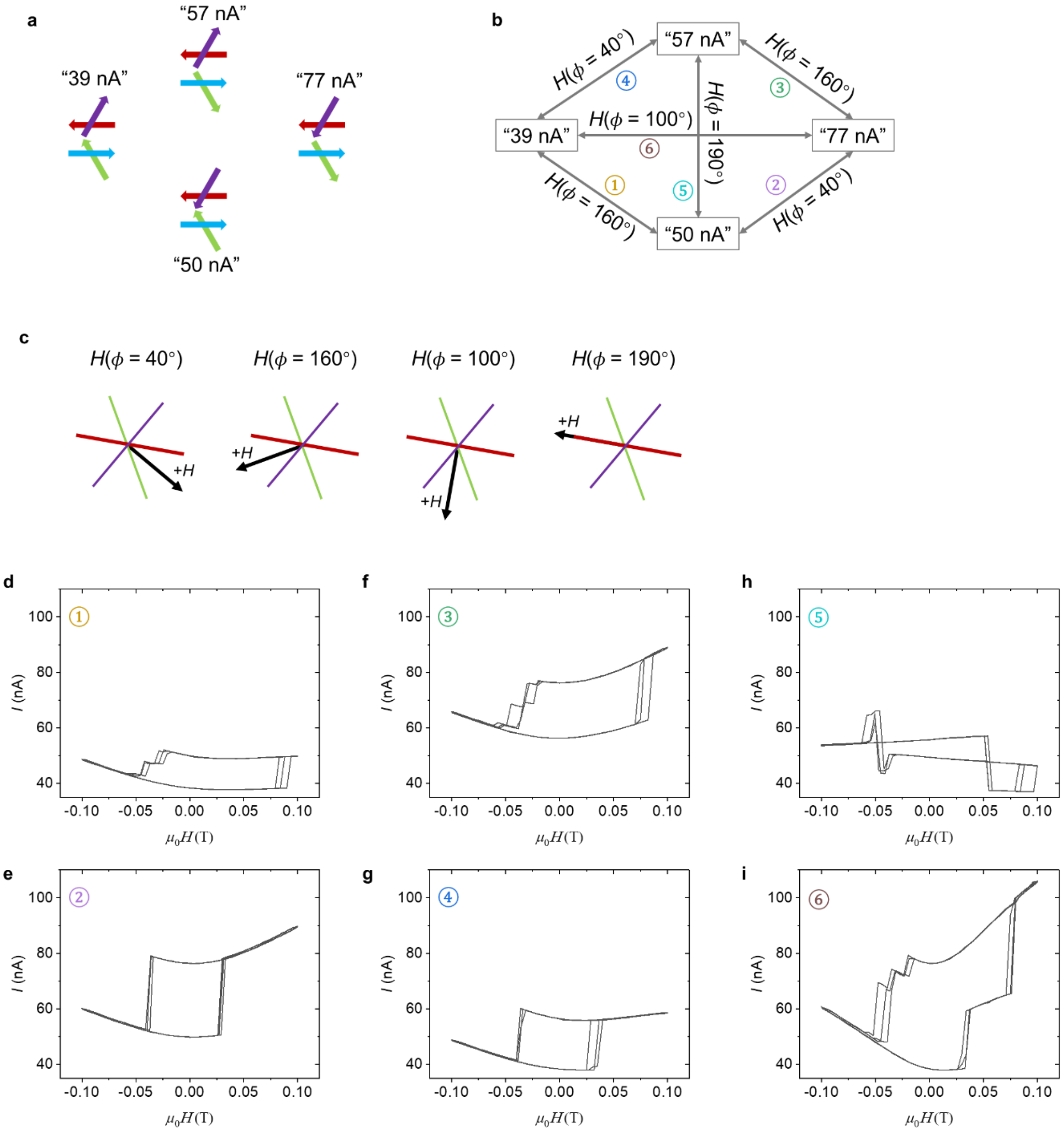

Supplementary Fig. 14 | Manipulating the four nonvolatile states with a flipped spin configuration in the CrSBr bilayer. a, In analogy to Fig. 3c of the main text, except the spin configuration in the CrSBr bilayer is flipped. **b**, Same as Fig. 4a of the main text. **c**, Same as Fig. 4b of the main text. **d-i**, Experimental demonstrations of the switching relationships in **b**. Three successive loops are used for each switching. ±0.1 T field sweep range and 20 mV DC bias are used.

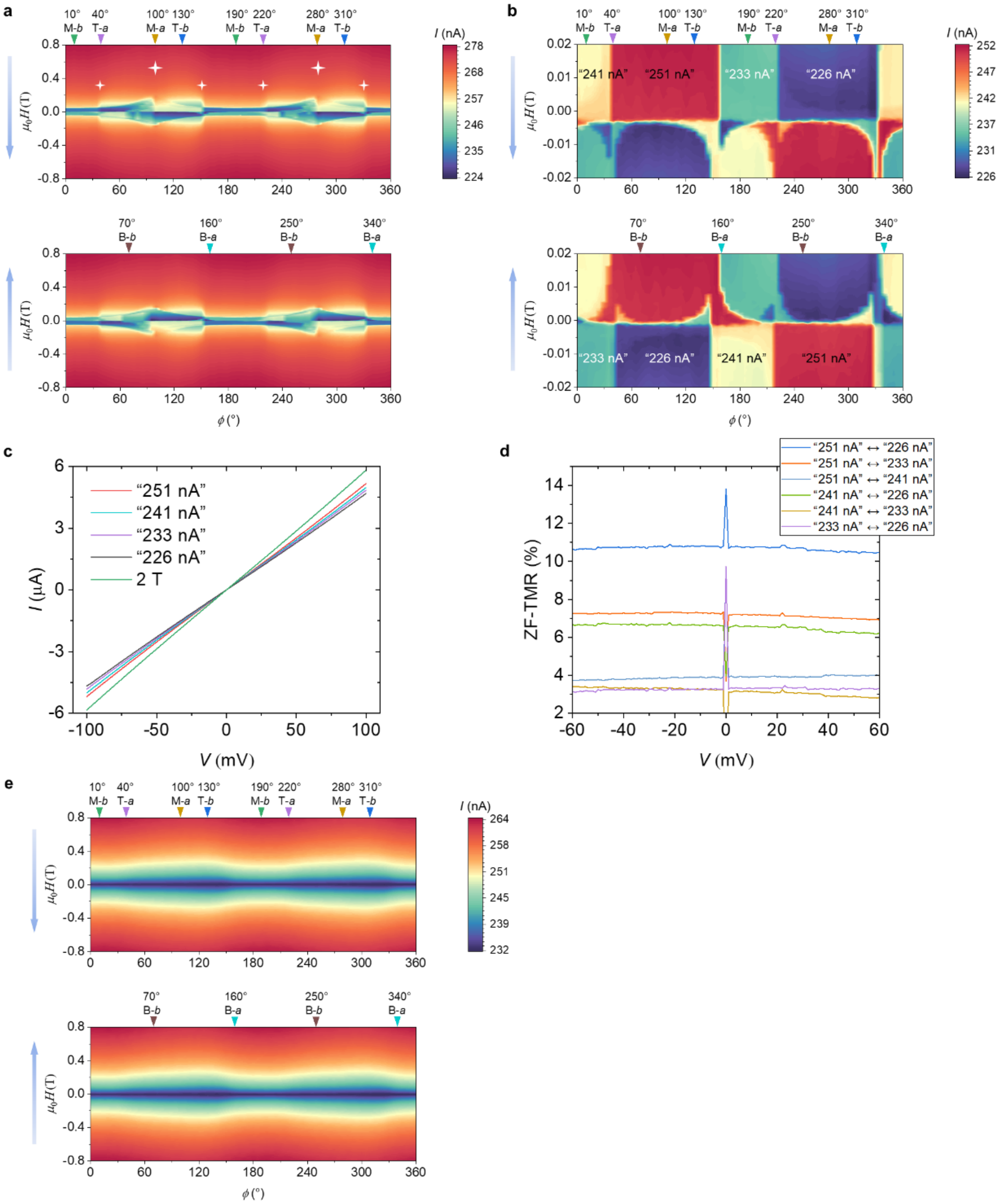

Supplementary Fig. 15 | High-temperature experimental results of the twisted CrSBr monolayer/bilayer/monolayer MTJ. a-b, Field orientation dependence of the tunneling current for field oriented within the *ab* plane. ±0.8 T field sweep range for **a**, ±0.02 T field sweep range for **b**, and 3 mV DC bias for both. **c**, *I*-*V* curves at ZF and 2 T. **d**, Calculated ZF-TMR ratio as a function of bias based on the ZF *I*-*V* curves of **c**. **a-d** are obtained at 120 K. **e**, Field orientation dependence of the tunneling current for field oriented within the *ab* plane. ±0.8 T field sweep range and 3 mV DC bias are used at 140 K.

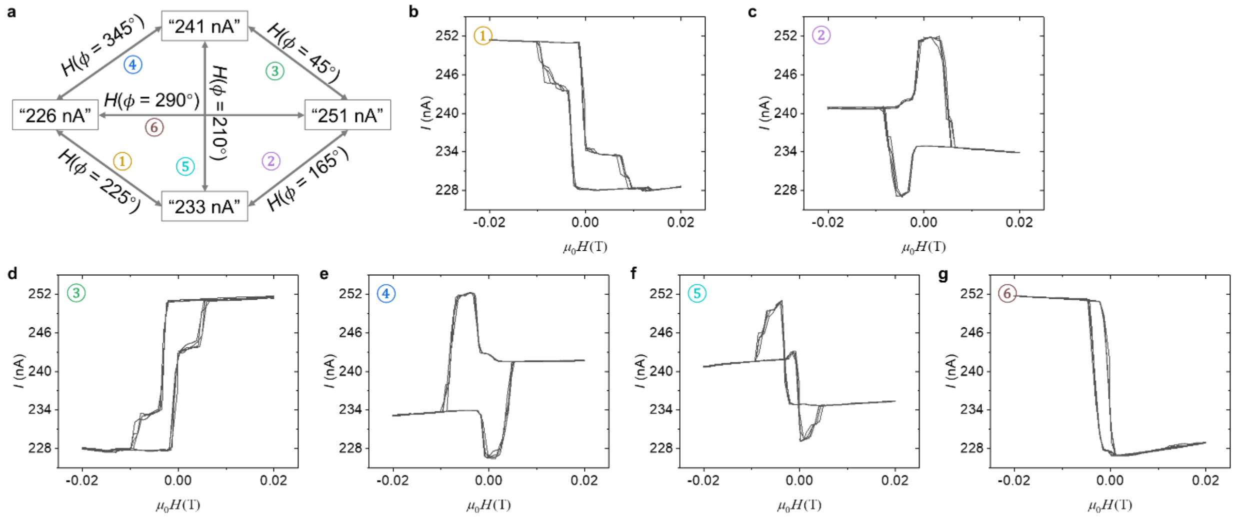

Supplementary Fig. 16 | Manipulating the four nonvolatile states in the twisted CrSBr monolayer/bilayer/monolayer MTJ at 120 K. a, Diagram of switching relationships among the four states connected by $H(\Phi)$. $H(\Phi)$ means the external field is oriented at Φ. There are six switchings, which are numbered. **b-g**, Experimental demonstrations of the switching relationships in **a**. Three successive loops are used for each switching. ±0.02 T field sweep range and 3 mV DC bias are used.

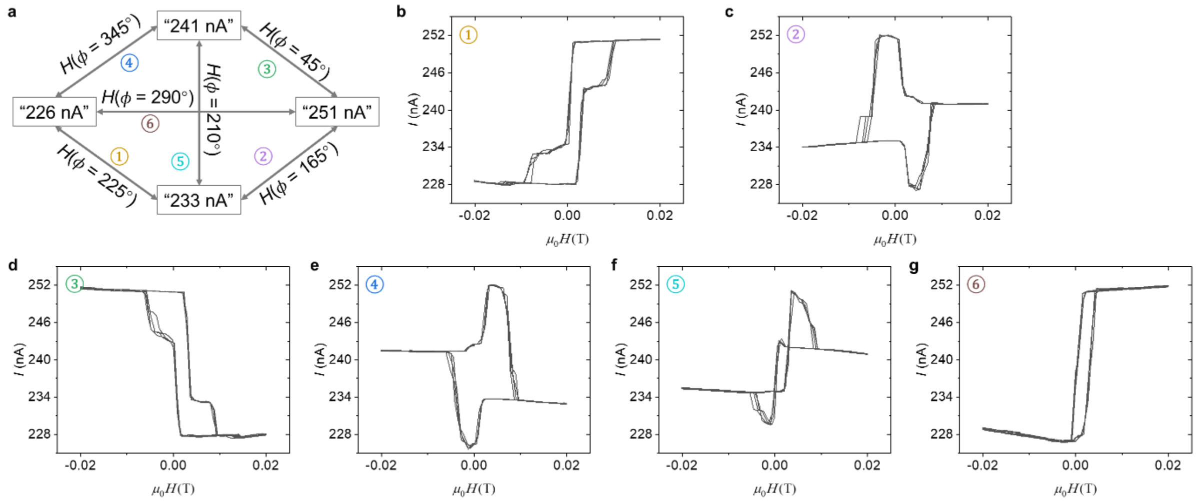

Supplementary Fig. 17 | Manipulating the four nonvolatile states in the twisted CrSBr monolayer/bilayer/monolayer MTJ at 120 K. Same as Supplementary Fig. 16, except the spin configuration in the CrSBr bilayer is flipped.

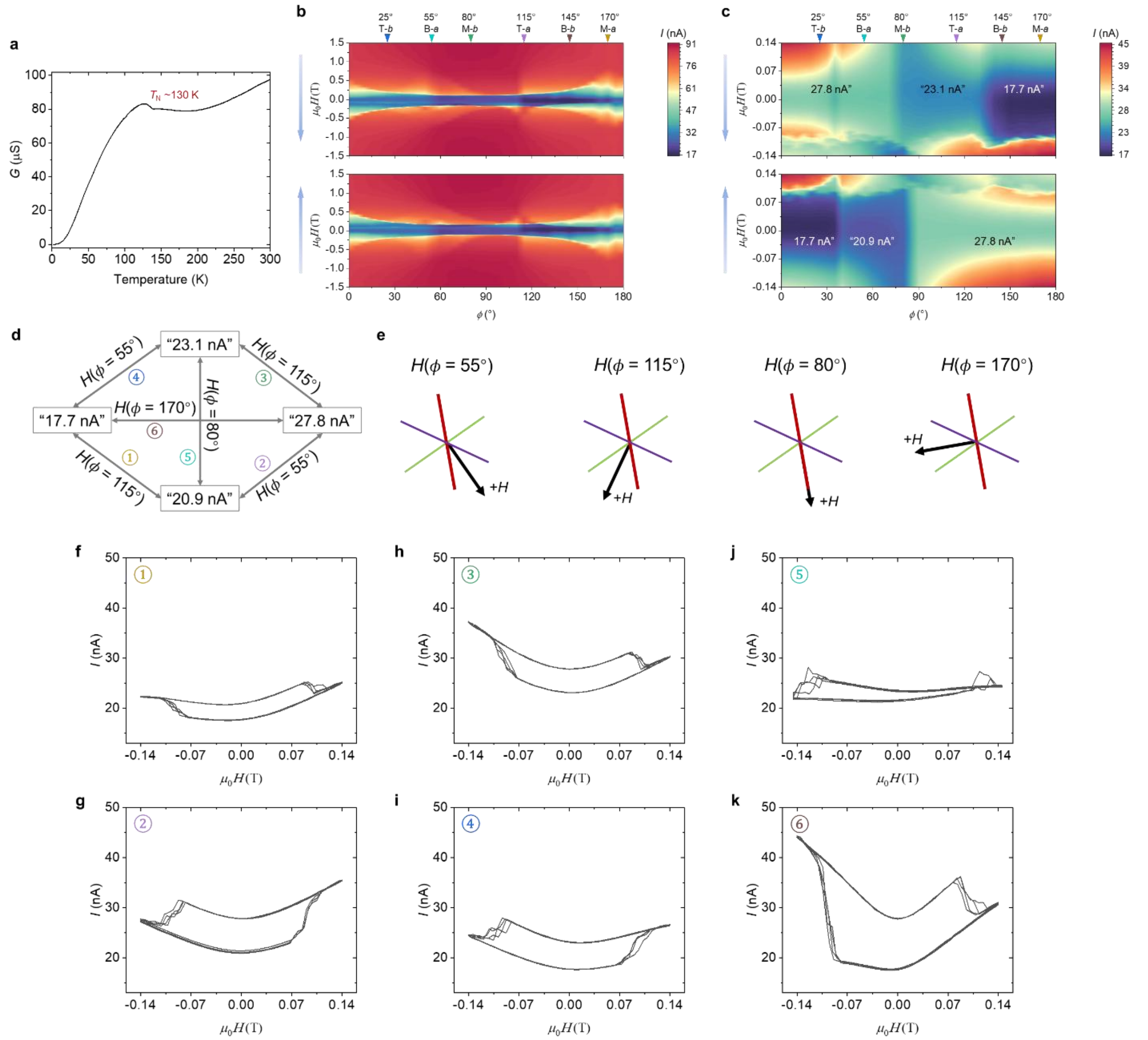

Supplementary Fig. 18 | Experimental results measured on the second twisted CrSBr monolayer/bilayer/monolayer MTJ. a, Conductance *versus* temperature at ZF. **b-c**, Field orientation dependence of the tunneling current for field oriented within the *ab* plane. ±1.5 T field sweep range for **b**, ±0.14 T field sweep range for **c**, and 30 mV DC bias for both. **d-k**, In analogy to Fig. 4 of the main text, manipulating the four nonvolatile states. Four successive loops are used for each switching of **f-k**, and ±0.14 T field sweep range and 30 mV DC bias are used.

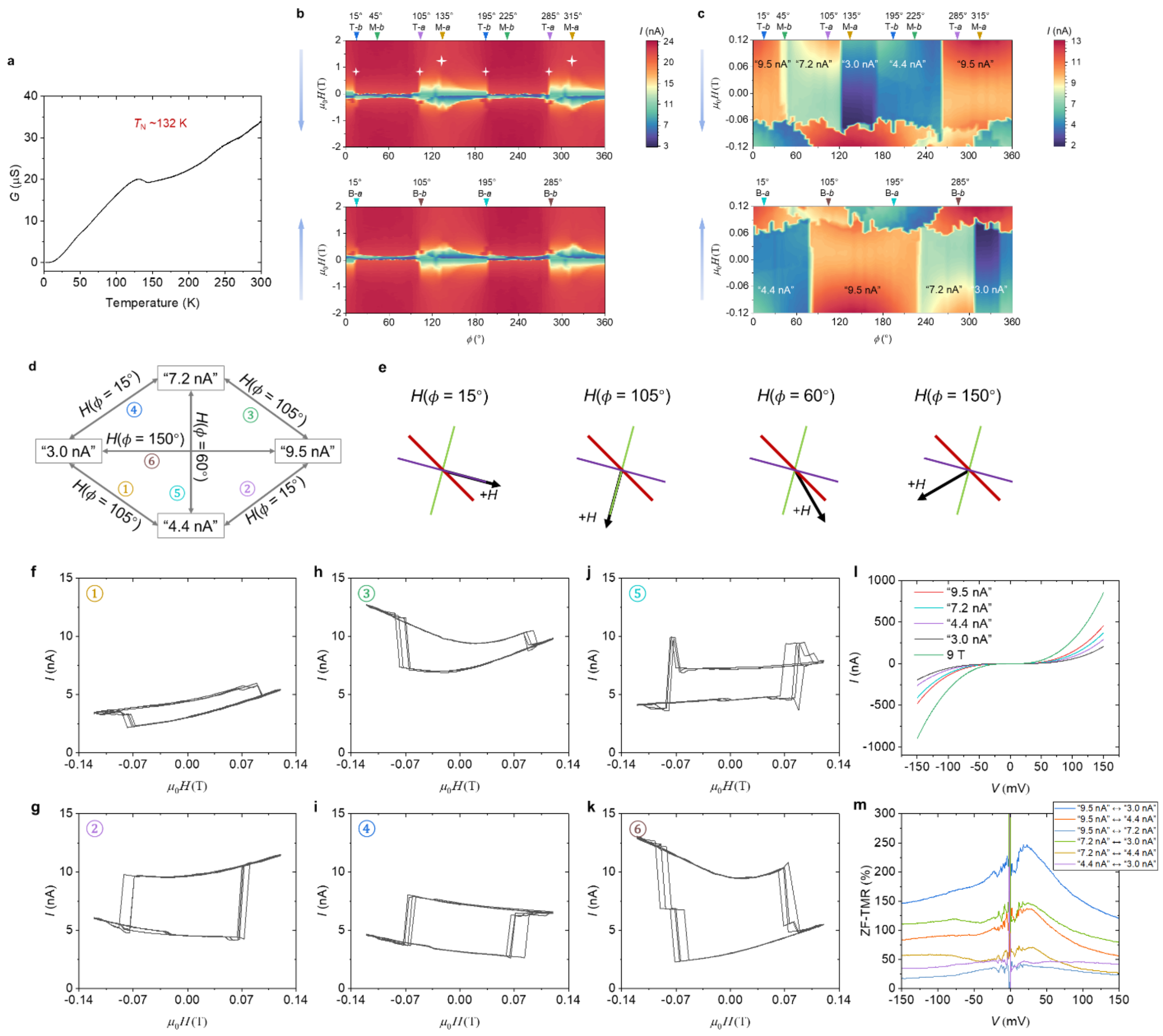

Supplementary Fig. 19 | Experimental results measured on the third twisted CrSBr monolayer/bilayer/monolayer MTJ. This MTJ has a 30° twisted top interface and a 60° twisted bottom interface. **a**, Conductance *versus* temperature at ZF. **b-c**, Field orientation dependence of the tunneling current for field oriented within the *ab* plane. ±2 T field sweep range for **b**, ±0.12 T field sweep range for **c**, and 40 mV DC bias for both. **d-k**, In analogy to Fig. 4 of the main text, manipulating the four nonvolatile states. Four successive loops are used for each switching of **f-k**, and ±0.12 T field sweep range and 40 mV DC bias are used. **l**, *I-V* curves at ZF and 9 T. **m**, Calculated ZF-TMR ratio as a function of bias based on the ZF *I-V* curves of **l**.

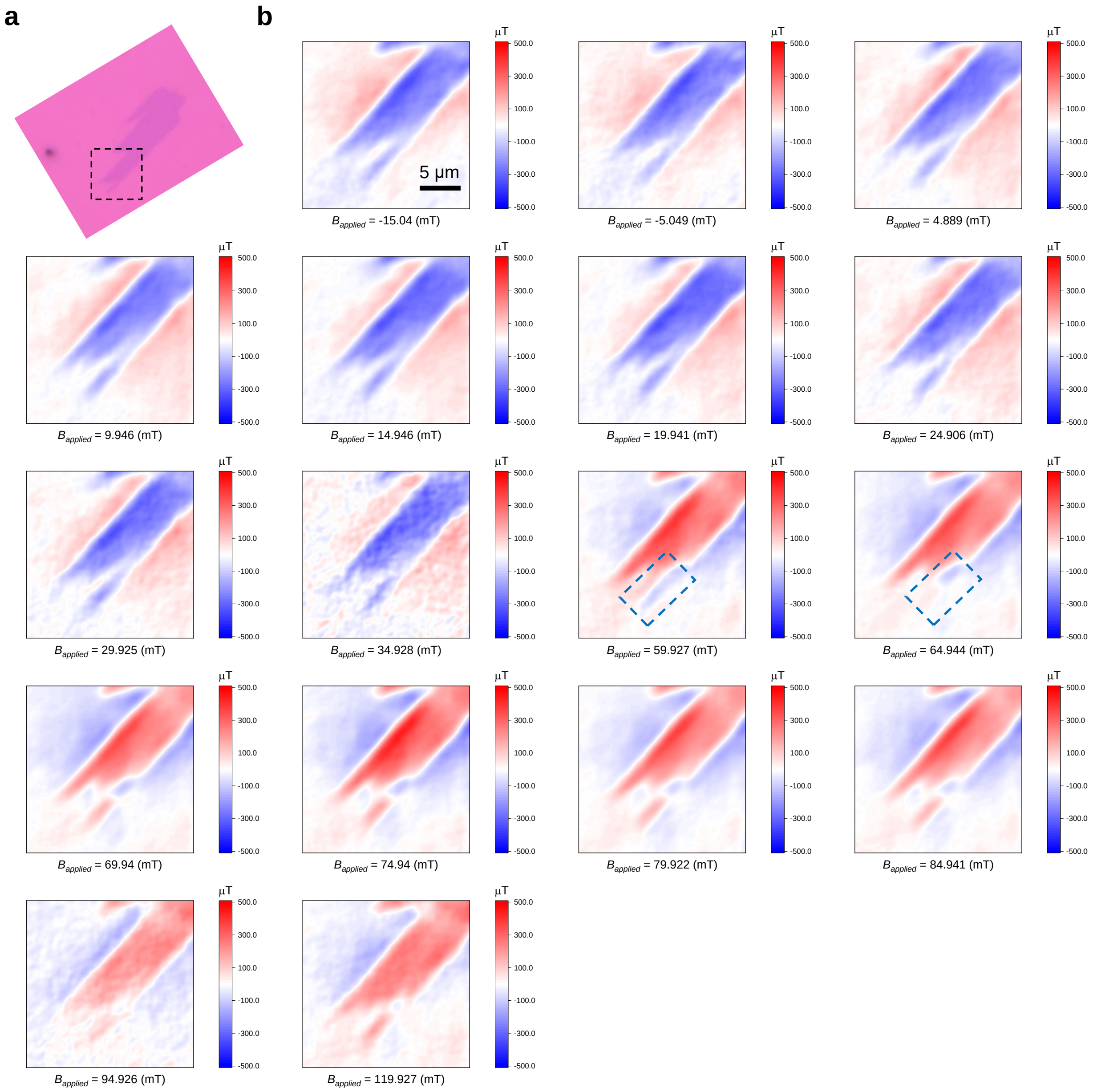

Supplementary Fig. 20 | Nitrogen-vacancy images of a CrSBr 1L device (fabricated by transfer) during the magnetization process. a, Optical image. The area outlined by the dashed line is measured by the nitrogen-vacancy microscope. **b**, The nitrogen-vacancy images at each applied field. Magnetic domains are marked by the blue dashed lines.

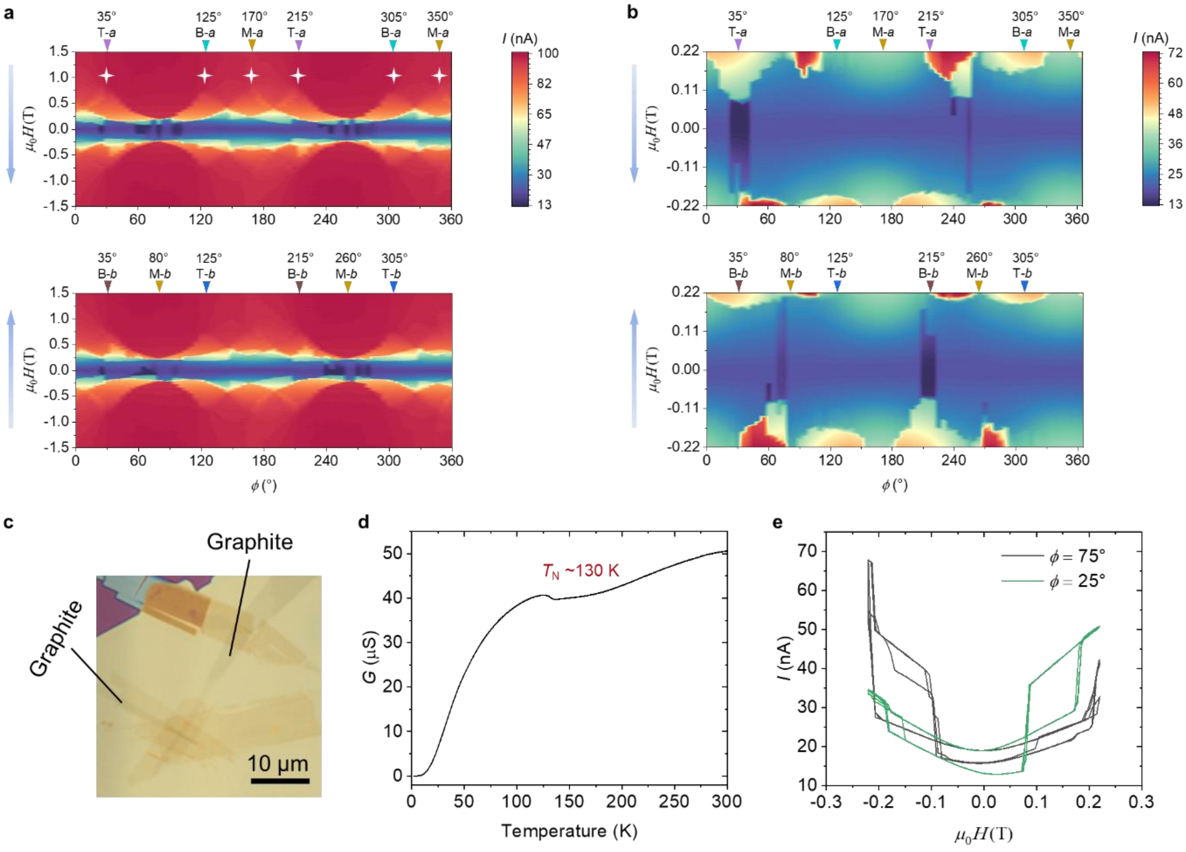

Supplementary Fig. 21 | Experimental results measured on a 45 ° twisted CrSBr bilayer/bilayer/bilayer MTJ. a-b, Field orientation dependence of the tunneling current for field oriented within the *ab* plane. ±1.5 T field sweep range for **a**, ±0.22 T field sweep range for **b**, and 60 mV DC bias for both. **c**, An optical image of the device. Note the relatively misaligned angle between the top bilayer and the bottom bilayer is 90°. **d**, Conductance *versus* temperature at ZF. **e**, Three successive loops are used for each switching. ±0.22 T field sweep range and 60 mV DC bias are used.

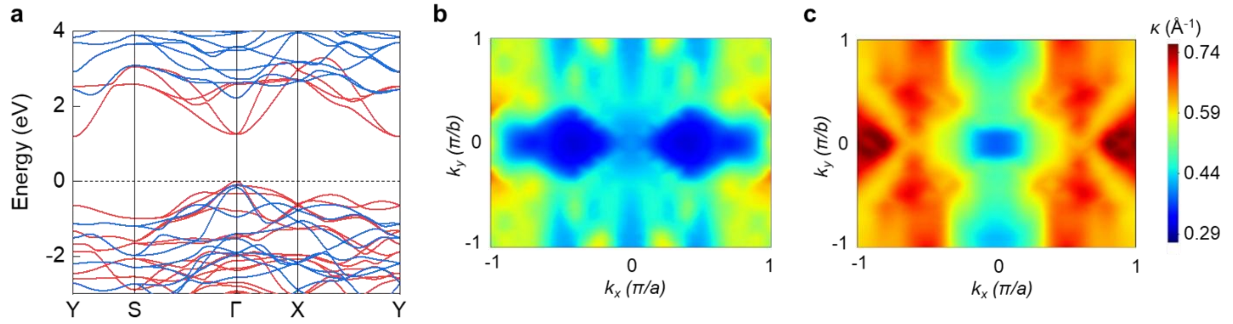

Supplementary Fig. 22 | Physical origin of the TMR effect. a, Energy band structure of CrSBr monolayer. The red curves represent the up-spin bands, and the blue curves represent the down-spin bands. The valence band maximum (VBM) is set to zero. **b-c**, Distribution of the lowest decay rates in the 2D Brillouin zone for spin-up (**b**) and spin-down (**c**) electrons at E_F.

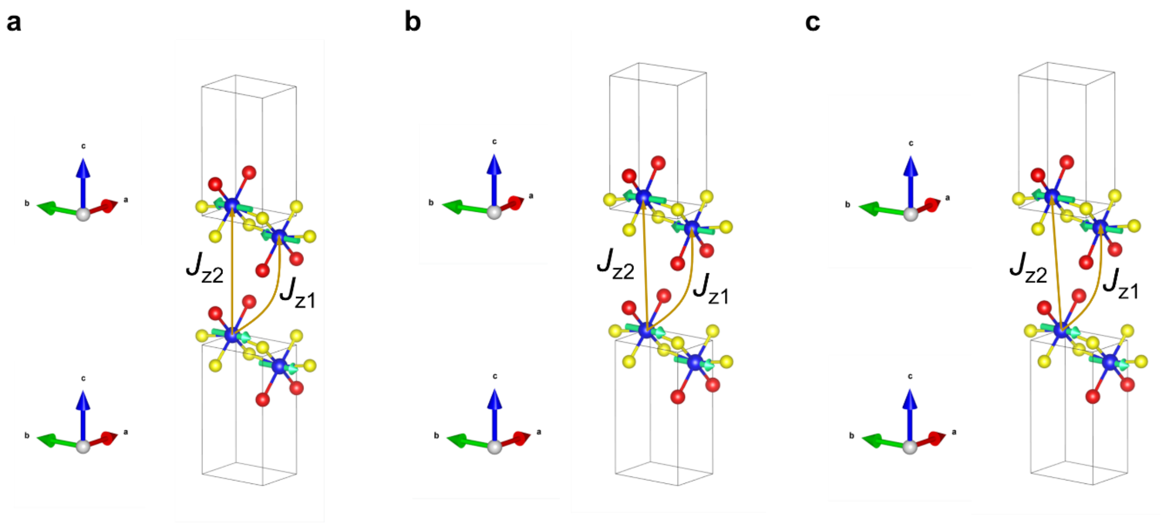

Supplementary Fig. 23 | Schematic of the interlayer magnetic exchange interactions. a-c, Interlayer exchange interactions of J_{z1} and J_{z2}. Untwisted (**a**), 5°-twisted (**b**), and 5°-twisted + 0.5 Å (**c**). The 0.5 Å shift is along the *b*-axis. Blue, yellow, and red balls correspond to Cr, S, and Br, respectively.

Supplementary Table 1 | Calculated interlayer exchange parameters (J_i in meV; see Supplementary Fig. 23) for CrSBr untwisted bilayer, twisted bilayers with 3° and 5°, and twisted bilayers with 3° + 0.5 Å and 5° + 0.5 Å. The 0.5 Å shift is along the *b*-axis. *a* = 3.5 Å.

	J_{z1}					J_{z2}				
c/a	**untwisted**	**3°**	**5°**	**3° + 0.5 Å**	**5° + 0.5 Å**	**untwisted**	**3°**	**5°**	**3° + 0.5 Å**	**5° + 0.5 Å**
2.266	-0.31	-0.254	-0.188	0.0007	0.0011	-0.01036	-0.0091	-0.0072	0.00031	0.00053
2.3	-0.312	-0.256	-0.191	0.00069	0.00103	-0.00878	-0.0076	-0.007	0.0003	0.00047
2.323	-0.314	-0.258	-0.194	0.00065	0.00091	-0.00845	-0.0071	-0.0067	0.00027	0.00045
2.345	-0.315	-0.26	-0.196	0.00058	0.00082	-0.00816	-0.0068	-0.0064	0.00026	0.00043
2.368	-0.317	-0.262	-0.198	0.00055	0.00081	-0.00788	-0.0063	-0.006	0.00023	0.00039
2.391	-0.319	-0.265	-0.2	0.00053	0.00078	-0.00764	-0.0058	-0.0058	0.0002	0.00034
2.413	-0.321	-0.268	-0.202	0.00049	0.00073	-0.00744	-0.0055	-0.0055	0.00015	0.00026

Supplementary Note 1. Effects of magnetic domains

An MTJ usually has two magnetic layers, i.e., a free layer and a reference layer. The magnetization of the reference layer is always pinned and only the magnetization of the free layer is tuned. In Supplementary Fig. 24a, suppose the gold layer is the free layer and the blue layer is the reference layer. In the left (right) panel of Supplementary Fig. 24a, the interlayer magnetization configuration between the free and reference layers is antiparallel (parallel). Suppose two domains appear in the free layer, see Supplementary Fig. 24b, in both left and right panels, the antiparallel and parallel interlayer configurations coexist, which would largely reduce the TMR. A salient example is that if the volume fraction of the two domains is 50% to 50%, the TMR is 0%. By contrast, a single domain in the free layer (Supplementary Fig. 24a) maximizes the TMR. All our devices show large TMR at ZF. Hence, we believe a single domain mechanism accounts for that, which is confirmed by the nitrogen-vacancy images shown in Supplementary Fig. 20.

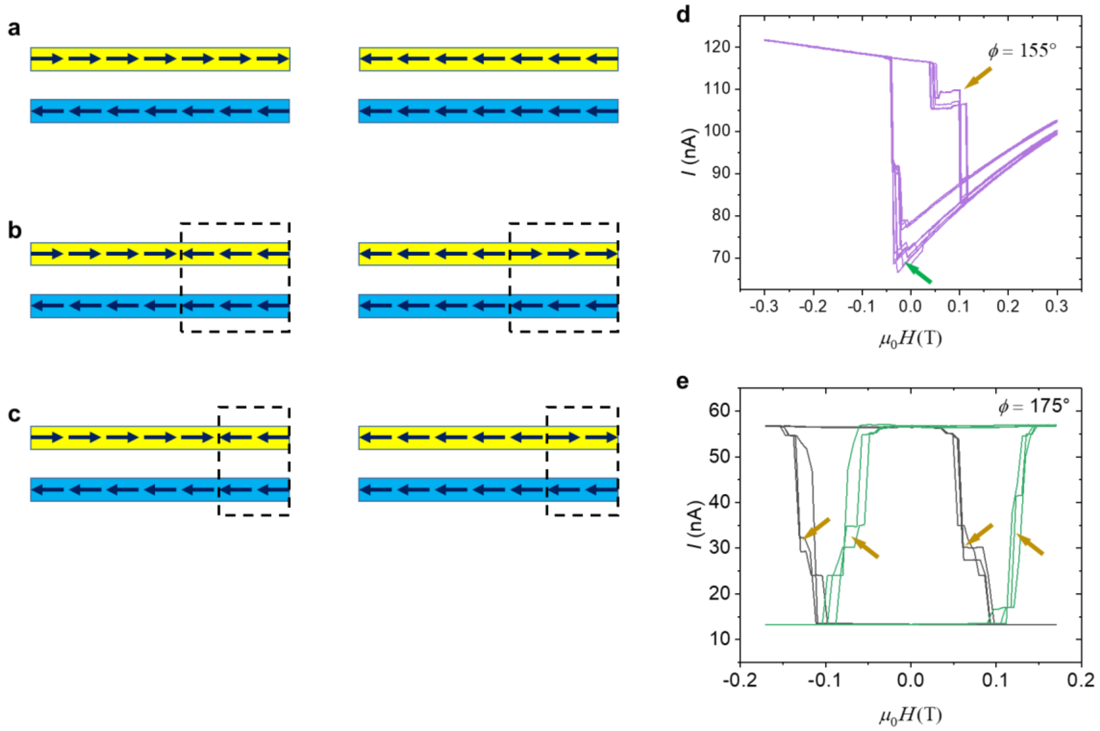

Supplementary Fig. 24 | The effects of magnetic domains. a-c, Schematics for the magnetoresistance of MTJs showing that domains in an MTJ would reduce the TMR. **d**, 10 *I-H* loops for a 55° twisted CrSBr 1L/1L MTJ. **e**, Same as Supplementary Fig. 10e. In **d-e**, the green arrow shows that domains appear at ZF and the gold arrows show that domains appear close to H_c. **d** is from Fig. S15f of *Nature 632.8027 (2024): 1045-1051*.

In addition, the nitrogen-vacancy images of the twisted CrSBr multiplayer stacks of 1L/1L, 1L/2L, and 2L/2L have been carefully investigated by our co-authors in their prior work (*arXiv:2410.19209, 2024*), in which the magnetic domains have been visualized at finite fields (e.g., a 1L/1L sample in Fig. 1e of *arXiv:2410.19209, 2024*). However, these magnetic domains switch completely into a single domain state using a suitably large field. Moreover, the magnetic domains not only appear in the overlapped portion of 1L/1L but also appear in the bare portion of 1L. Thus, we think the demagnetizing field inside one 1L (see Methods) and the stray field from other 1L (see Supplementary Note 2) are not the main reasons accounting for the magnetic domains. Supplementary Fig. 20 is the data of a single 1L device in our work, which also shows domains (e.g., marked by the blue dashed lines) but fewer than Fig. 1e of *arXiv:2410.19209, 2024*. We think the reason is that more transferring procedures used to fabricate the 1L/1L stack result in more strain. Both the results in our work and in their prior work indicate that a single magnetic domain state can be initialized and maintained at zero field even if strains were introduced during the fabrication. This is also in accordance with the results of *Nano Letters 24.41 (2024): 13068-13074*.

However, it should be pointed out that the formation of domains can alter the interlayer magnetization configurations between the free layer and reference layer, but with thermal randomness usually accompanied. For example, suppose the two domains in Supplementary Fig. 24b appear at a certain field, if changing the field and then going back to the original field, the domains usually cannot be restored because of thermal randomness (suppose become Supplementary Fig. 24c), whose magnetoresistances differ from that of the configurations shown in Supplementary Fig. 24b. Such randomness has been observed in our experiments. In our previous work (*Nature 632.8027 (2024): 1045-1051*), we measured the *I-H* results of a twisted CrSBr 1L/1L MTJ by repeatedly sweeping the field loops. The different currents randomly appear at ZF (green arrow of Supplementary Fig. 24d) due to the random interlayer magnetization configurations. More commonly, since spin-flipping is a critical phenomenon that is susceptible to thermal fluctuations, strains and defects, domains usually form adjacent to H_c, which also manifest themselves as random currents (gold arrow of Supplementary Fig. 24d). The random domains formed close to H_c are also frequently observed in our current devices, for example, see Supplementary Fig. 24e, but we do not see any randomness at ZF. Hence, again, the formed domains have nothing to do with our ZF NV, which only change the details of spin-flipping. It should be further pointed out that such details vary over devices because strains and defects cannot be well controlled by manual device fabrication. Thus, we note that any attempt to extract some general laws from the experimental data, especially related to magnetic domains, should carefully consider device differences, thermal randomness and reproducibility (*Nature Materials 23.2 (2024): 212-218*; *Advanced Materials 37.8 (2025): 2415774*).

Supplementary Note 2. Applicability prospects of 2D twisted MTJs

1. Scaling. Our present work and previous work (*Nature 632.8027 (2024): 1045-1051*) demonstrate that ZF NV constantly emerges in the twisted MTJs, varying the twist angle from 3° to 80°. In stark contrast to other twist systems, a particular twisted angle is usually needed. For example, superconductivity arises in the magic-angle (1.1°) twisted graphene bilayer (*Nature 556.7699 (2018): 43-50*). Once missing 1.1° slightly, superconductivity disappears. We believe that the flexibility of the twisted angle makes 2D twisted MTJs realistically achievable at scale. We can anticipate two primary strategies for fabricating them. (a) After large-scale growths of 2D magnetic monolayer and bilayer by e.g., CVD, ALD, MBE, large-sized samples can be brought together to form twisted structures by large–scale transfer techniques (e.g., *Nature Electronics 7.2 (2024): 96-97*; *Nature Electronics 7.2 (2024): 119-130*; *Nature communications 13.1 (2022): 4409*; *Nature Electronics (2025): 1-13*). Because a strict twisted angle is not necessary for 2D twisted MTJs, it is tolerable that the twisted angle fluctuates over space. (b) Self-alignment strategies rely on thermodynamic equilibrium during the growth process. Other 2D twisted systems need a particular twisted structure with a strict twisted angle, but such a twisted structure is usually not thermodynamically favorable. By contrast, the twist angle can be used from 3° to 80° for 2D twisted MTJs. Thus, there could be some thermally stable or metastable twisted structures.

2. Operation. At present, as two early reports, our previous work (*Nature 632.8027 (2024): 1045-1051*) and present work only use an external magnetic field to write information. The next plan is to electrically write information using spin-transfer torque (STT) and spin–orbit torque (SOT), especially the latter, which has better durability. Thow Min Jerald Cham et al recently reported the SOT-induced magnetic resonance in the $PtTe_2$/CrSBr bilayer system (*Science (2025): eadq8590*). Their results indicate that SOT only diffuses into the CrSBr monolayer contacted by the $PtTe_2$ without influencing the other monolayer. Hence, two SOT electrodes contacting the top and bottom monolayers of a 1L/2L/1L MTJ, respectively, can serve as two separate write channels for independent control.

Science (2025): eadq8590 and *Physical Review B 111.2 (2025): 024410* report the resonant frequencies of 18 GHz to 35 GHz for the CrSBr multilayer, corresponding to 28 ps to 55 ps. We have not seen any related reports for the CrSBr monolayer. We estimate that the speed should be slightly slower since the CrSBr monolayer is ferromagnetic but with strong intralayer exchange interactions and strong magnetic anisotropy. Furthermore, the concepts of twisted MTJs are general. We anticipate that other faster 2D magnets can be used to enhance the operation speed in the future.

3. Miniaturization. Our 2D twisted MTJs are down to the atomic thickness, but the typical tunnel area is < 1 μm^2. Such lateral size can be further reduced. However, shrinking the size would eventually drive the

device into the superparamagnetic regime. Specifically, when the thermal fluctuation $k_B T$ is comparable to the action energy KV, the system becomes superparamagnetic. k_B is the Boltzmann constant, T is temperature, K is a constant which quantifies the energy density associated with magnetic anisotropy, and V is the volume of the magnet. Our experimental results in Fig. 2b suggest that the minimal H_c of the CrSBr monolayer is ~500 Oe (suppose this value originates from the magnetocrystalline anisotropy of the CrSBr system). Suppose every Cr carries $3\mu_B$ magnetic moment, where μ_B is the Bohr magneton. Finally, we obtain the critical size of ~6.68 nm^2 for the CrSBr monolayer at 2 K, which corresponds to 40 unit cells. The measured properties of the CrSBr monolayer could not be preserved in such a small sample but this estimated critical size manifests that our 2D twisted MTJs can be thermally stable down to a very small size. The estimated critical size is ~400 nm^2 at 120 K, considering the magnetocrystalline anisotropy is preserved.

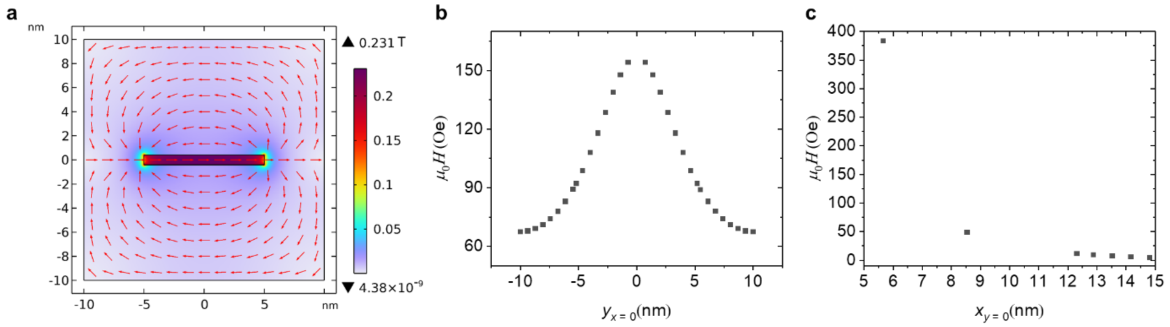

Supplementary Fig. 25 | Stray field simulation. a, Spatial distribution of stray fields produced by a CrSBr monolayer. **b**, The out-of-plane stray fields extracted from **a** at $x = 0$. **c**, The in-plane stray fields extracted from **a** at $y = 0$.

In contrast to the stray field-free in all-AF 2L/2L and 2L/2L/2L structures, the ferromagnetic CrSBr 1L in the 2L/1L and 1L/2L/1L structures would produce stray fields. Closely packing many MTJs could lead to crosstalk between them. We simulated the spatial distribution of stray fields produced by a CrSBr 1L (Supplementary Fig. 25a). The 1L/2L/1L stack can be treated as two monolayers separated by a bilayer, that is, a distance of ~1.4 nm (the bilayer thickness). At this distance, the stray field is ~140 Oe (Supplementary Fig. 25b). Note that this stray field is non-negligible, which could be the reason that the loops in Fig. 4 of the main text show exchange-bias–like shifts. However, the coercive field of the CrSBr monolayer is >500 Oe, which is far beyond the stray field. Moreover, we stack 1L/2L/1L along the z-direction. It is a 3D strategy. If one more monolayer is stacked along the z-direction (1L/2L/1L/2L/1L, note we do not show this because manually stacking more layers is very technologically difficult), the stray field would be ~280 Oe lower than the coercive field. We further evaluate the in-plane distribution. Supplementary Fig. 25a shows strong stray fields around the two terminals of the CrSBr sheet, but they

decay quickly along the *x*-direction, for example, ~50 Oe at a separated distance of 3.5 nm (Supplementary Fig. 25c).
