# Supplementary material for: Twisted atomic magnetic tunnel junctions with multiple nonvolatile states
Source: Nat Commun. 2026 Mar 12;17:2439. doi: 10.1038/s41467-026-70239-z (PMC12988127; doi:10.1038/s41467-026-70239-z)
Supplement: Supplementary file 3 — Description of Additional Supplementary Files [file 41467_2026_70239_MOESM3_ESM.pdf]

<u>Description of Additional Supplementary Files</u>

Supplementary Movie 1: Sweep field between ±1 T along the easy axis of the bottom CrSBr bilayer.

The denotations of the arrows are in line with the manuscript. The spin of the top monolayer rotates away from the external field orientation while reducing the external field from 1 T due to the uniaxial in-plane anisotropy. Then, a spin flip occurs in the bilayer due to interlayer antiferromagnetic interaction. At zero field, the spin of the top monolayer is aligned along the easy axis. While increasing the external field along the negative orientation, it is found that spin-flips first occur in the monolayer and then in the bilayer. Continuing to increase the field forces the spin of the top monolayer to rotate to the external field orientation. Similar processes happen while sweeping the field from -1 T to 1 T. Note that each spin heads to the opposite orientation at zero field determined by the forward/backward sweeping field.

Supplementary Movie 2: Sweep field between ±1 T along the easy axis of the top CrSBr monolayer.

The denotations of the arrows are in line with the manuscript. While reducing the external field from 1 T, the spins of the bottom bilayer first rotate away from the external field orientation together due to the uniaxial in-plane anisotropy and then a spin flip occurs due to interlayer antiferromagnetic interaction. At zero field, the spins of the bottom bilayer are antiferromagnetically aligned along the easy axis. While increasing the external field along the negative orientation, it is found that spin-flips first occur in the monolayer and then in the bilayer. Continuing to increase the field forces the spins of the bottom bilayer to rotate to the external field orientation. Similar processes happen while sweeping the field from -1 T to 1 T. Note that each spin heads to the opposite orientation at zero field determined by the forward/backward sweeping field.
